# Annealing temperature-dependent induced supramolecular chiroptical response of copolymer thin films studied by pump-modulated transient circular dichroism spectroscopy

**DOI:** 10.1038/s41598-024-63126-4

**Published:** 2024-06-03

**Authors:** Domenic Gust, Mirko Scholz, Vivien Schumacher, Jean-Christophe Mulatier, Delphine Pitrat, Laure Guy, Kawon Oum, Thomas Lenzer

**Affiliations:** 1 Faculty IV: School of Science and Technology, Department Chemistry and Biology, Physical Chemistry 2, University of Siegen, Adolf-Reichwein-Str. 2, 57068 Siegen, Germany; 2 Univ. Lyon, ENS de Lyon, CNRS UMR 5182, Laboratoire de Chimie, Université Claude Bernard Lyon 1, 69342 Lyon, France

**Keywords:** Circular dichroism, Reaction kinetics and dynamics, Excited states, Optical materials

## Abstract

Copolymer thin films showing induced supramolecular chirality are of considerable interest for optoelectronic applications such as organic light-emitting diodes. Here, we introduce a new helicene-like chiral additive with two octyloxy substituents which displays excellent chiral induction properties in an achiral polyfluorene copolymer, leading to a circular dichroism (CD) response of up to 10,000 mdeg. This chiral inducer also displays very good thermal stability, which enables us to perform an extended study on the induced chiroptical properties of the cholesteric copolymer thin films annealed at different temperatures in the range 140–260 °C. Starting from about 180 °C, a distinct change in the morphology of the CD-active film is observed by CD microscopy, from micrometre-size granular to extended CD-active regions, where the latter ones display skewed distributions of the dissymmetry parameter *g*_abs_. Broadband Müller matrix spectroscopy finds a pronounced CD and circular birefringence (CB) response and only weak linear dichroism (LD, LD’) and linear birefringence (LB, LB’). Ultrafast transient CD spectroscopy with randomly polarised excitation reveals a clean mirror-image-type transient response, which shows a second-order decay of the S_1_ population due to singlet–singlet annihilation processes.

## Introduction

**Figure 1:**
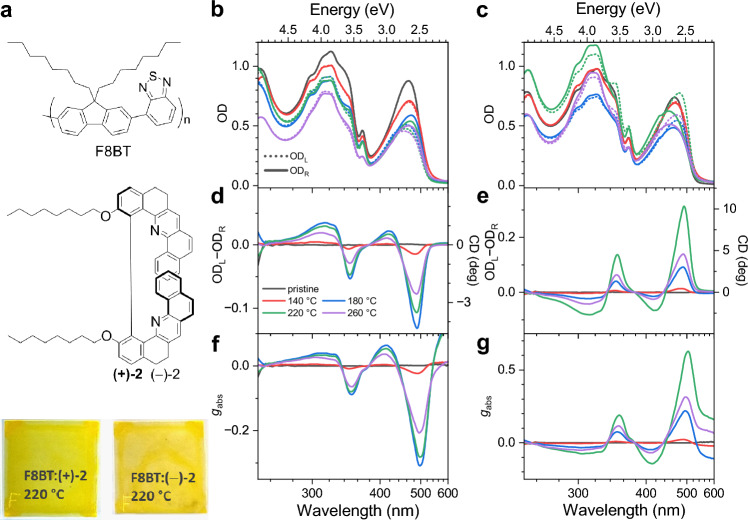
Steady-state absorption and CD response of F8BT:( +)-2 and F8BT:( −)-2 thin films. (**a**) Chemical structure of the F8BT copolymer (1) and the helicene-like chiral inducers (2) including photographs of two thin films annealed at 220 °C on glass (front view). (**b**) Absorption spectra for left-circularly polarised (dotted lines) and right-circularly polarised light (solid lines) of F8BT:( +)-2 thin films: pristine (black), *T*_ann_ = 140 °C (red), 180 °C (blue), 220 °C (green) and 260 °C (violet). (**c**) Same as in panel b, but for F8BT:( −)-2 films. (**d** and **e**) Corresponding CD spectra of the thin films obtained from the absorption spectra in panels b and c, respectively. The CD response in degree is shown on the right *y*-axis. (**f** and **g**) Corresponding dissymmetry parameters *g*_abs_ obtained from the absorption spectra in panels b and c, respectively.

Thin films of copolymers with chiral supramolecular structure have attracted considerable attention due to their unique chiroptical properties, typically reflected in a large circular dichroism and strongly circularly polarised luminescence (CPL)^1,2^. These properties make them attractive for a range of applications in organic optoelectronics, such as photodetectors^3,4^ or organic light-emitting diodes (OLEDs) that produce circularly polarised luminescence^5–7^. Several research groups have successfully synthesised and investigated such supramolecular structures by using intrinsically chiral copolymers^8–18^. Another way to achieve a supramolecular chiroptical response is by embedding a chiral inducer into an achiral copolymer. Typical systems previously investigated are blends of the copolymer poly-[(9,9-di-*n*-octylfluorenyl-2,7-diyl)-*alt*-(benzo[2,1,3]thiadiazol-4,8-diyl)] (shortly F8BT, compound 1 in Fig. 1a) and various chiral molecular additives, such as 1-aza[6]helicene, limonene, or substituted binaphthalene derivatives^19–22^. Recently, helicene-like molecules based on dibenzo[*c*,*h*]acridine moieties, ( +)- and ( −)-2,2′-dimethoxy-5,5′,6,6′-tetrahydro-1,1′-bidibenzo[*c*,*h*]acridine and a methylene-bridged analogue, were synthesised^23^, which showed significant potential for achieving a strong CD and CPL response^24^. Especially in this system, substantial chiral induction occurred in F8BT, with an absolute CD response (difference of the optical density (OD) for left circularly polarised (L or LCP) and right circularly polarised (R or RCP) light, i.e. OD_L_ − OD_R_) of about 10,000 mdeg and a dissymmetry parameter *g*_abs_ = (OD_L_ − OD_R_)/(0.5·(OD_L_ + OD_R_)) of up to 0.5 were obtained^24^. The strong CD response is of supramolecular origin and likely arises from the formation of a cholesteric phase^24^. A steep increase of *g*_abs_ was observed with increasing film thickness up to about 150 nm and a saturation behaviour above. Such a thickness dependence of *g*_abs_ for cholesteric arrangements is well described by the dielectric-tensor model of Lakhwani and Meskers^25^.

The structure of the chiral helicene-like inducers can be conveniently tuned by modifying the ring systems constituting the helicene-like part or by applying further modifications by means of a variation of the substituents on the aromatic rings^23,26,27^. In this way, the rigidity of the helicene-like system and its molecular interaction with the polymer environment can be widely adjusted. Yet, the impact of these modifications on the chiral induction properties of these additives have not been sufficiently explored so far. In this work, benefiting from a long experience in the synthesis and the study of the chiroptical properties of enantiopure extended binaphthol-like compounds and their derivatives^26–28^, the helicene-like molecules ( +)- and ( −)-2,2′-dioctyloxy-5,5′,6,6′-tetrahydro-1,1′-bidibenzo[*c*,*h*]acridine (Fig. 1a, shortly denoted as ( +)-2 and ( −)-2) were newly synthesised. They were decorated with two *n*-octyloxy substituents to allow for an optimal incorporation in thin film blends of F8BT. The impact of this chemical modification on the induction of supramolecular chirality in F8BT is investigated here in detail for spin-coated F8BT:( +)-2 and F8BT:( −)-2 thin film blends. In particular, the influence of the annealing temperature on their chiroptical response is studied. A complete chiral characterisation is achieved by a combination of different steady-state chiroptical methods, such as CD spectroscopy and CD microscopy^24,29^ and a newly developed setup for broadband Müller matrix spectroscopy. In addition, we present here ultrafast transient CD (TrCD) experiments with femtosecond time resolution to study the ultrafast response of these thin-film systems by employing a new polarisation modulation scheme for the electronic excitation of these thin-film systems to arrive at an artefact-free detection of the TrCD signal.

## Results and discussion

### Steady-state absorption and CD spectroscopy of F8BT: ( +)-2 and F8BT: ( −)-2 thin film blends

The panels b and c of Fig. 1 summarise the optical absorption spectra of F8BT:( +)-2 and F8BT:( −)-2 thin films, respectively, which were either kept in pristine form (black) or processed at an annealing temperature (*T*_ann_) of 140 °C (red), 180 °C (blue), 220 °C (green) or 260 °C (violet) for 15 min. Dotted and solid lines refer to OD_L_ and OD_R_, respectively. F8BT displays a pronounced S_0_→S_1_ absorption band, which is centred at about 465 nm, and features additional partially overlapping electronic absorption bands below 360 nm with a peak centred at about 320 nm. The chiral additives ( +)-2 and ( −)-2 show a characteristic sharp absorption peak at 368 nm. The thermal annealing step results in a slightly reduced absorption amplitude and a broadened S_0_→S_1_ absorption band, indicating a structural rearrangement and consequently changed absorption properties of the blends. In addition, the films annealed at 260 °C show a slightly reduced absorption band of the chiral additive, which might indicate some thermal decomposition of the inducer at that temperature. The resulting CD spectra for the thin films are shown in panels d and e, respectively. In the case of the F8BT:( +)-2 blends (panel d), CD activity is only found starting from annealing temperatures of at least 140 °C (red). This is attributed to the crystallisation from an amorphous glassy state to a chiral nematic phase or rubbery state^24,30^. The maximum CD signal is observed for *T*_ann_ = 180 °C (blue) and then decays slightly at 220 °C and 260 °C. Pronounced CD bands are observed at 490, 420, 345 and 315 nm. For the F8BT:( −)-2 blends (panel e), the CD spectra resemble the mirror images of those in panel f. Here, the largest CD activity of about 10 deg is observed for the blend annealed at a temperature of 220 °C. It is not clear yet, why the temperatures for reaching the peak CD values are different for the blends with chiral additives of opposite chirality. Possibly, there is a plateau temperature region for maximum CD activity in the range 180–220 °C, and subtle changes in the annealing conditions are responsible for the variations. We note that, in contrast to the absorption spectra, there are no visible CD contributions from the chiral inducers in the CD spectra, which should appear close to their absorption band at 368 nm.

The spectra of the dissymmetry parameter *g*_abs_ in panels f and g closely resemble the corresponding CD spectra, and a peak value of about 0.6 is observed. The results suggest that the helicene-like additives ( +)-2 and ( −)-2 are efficient chiral inducers which can rival the performance of their previously studied analogues with two methoxy groups^24^. At the same time, they significantly exceed the chiral induction properties of rigid helicene-like inducers with a diether bridge connecting the two aromatic systems^24^.

### CD microscopy of F8BT:( +)-2 and F8BT:( −)-2 thin film blends with diffraction-limited resolution

**Figure 2:**
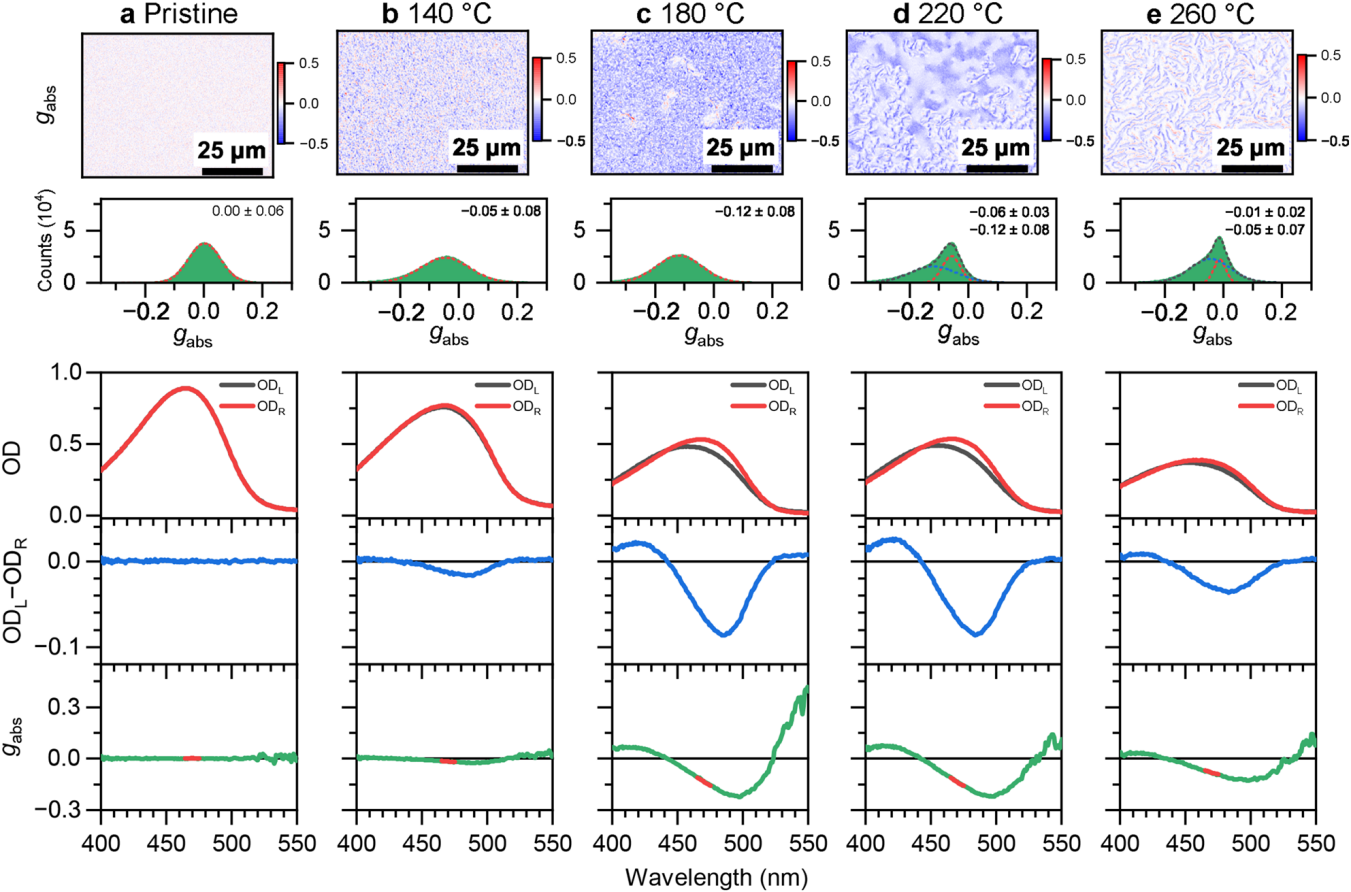
CD microscopy of F8BT:( +)-2 blends. (**a**) Microscope image (80 × 60 μm^2^, length of scale bar: 25 μm) showing the dissymmetry parameter *g*_abs_ of a F8BT:( +)-2 thin film (top) with the distribution of *g*_abs_ values (middle) including a Gaussian fit (red dashed line), determined over the entire field of view, and corresponding spectra integrated over the entire field of view (210 × 160 μm^2^, three panels at the bottom) displaying OD_L_ (black line) and OD_R_ (red line), the CD spectrum (OD_L_ − OD_R_, blue line) and the *g*_abs_ spectrum (green line), with the thick red line indicating the spectral region selected by the bandpass filter (470 nm, FWHM 10 nm) used for CD imaging. This thin film was not annealed (pristine). (**b–e**) Same as in panel a, but for F8BT:( +)-**2** thin films annealed at temperatures *T*_ann_ of 140 °C, 180 °C, 220 °C and 260 °C, respectively. The *g*_abs_ distributions for the films annealed at 220 °C and 260 °C must be fitted by a sum of two Gaussian functions (red and blue dashed lines), with the sum represented by a black dashed line.

**Figure 3:**
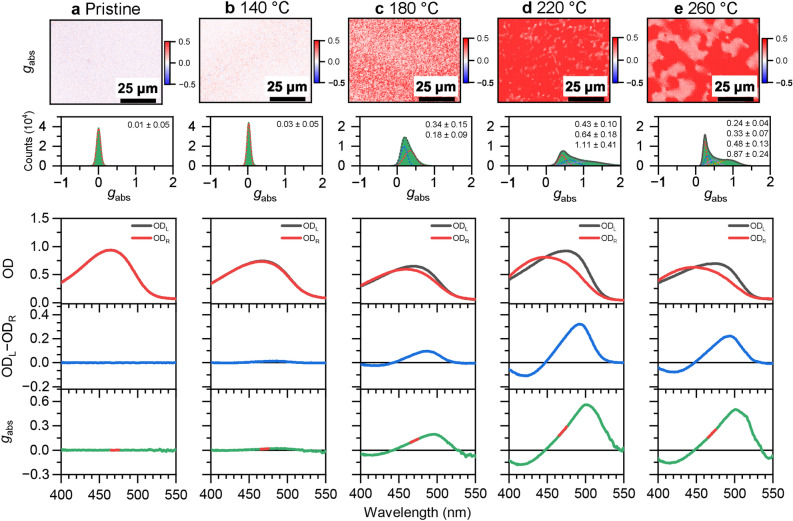
CD microscopy of F8BT:( −)-2 blends. Same as in Fig. 2, but for the F8BT thin film blends employing the helicene-like inducer ( −)-2 having opposite chirality.

To further characterise the chiroptical properties of these thin films as a function of annealing temperature, CD microscopy was carried out on a setup which we previously used for studying other polyfluorene-based copolymer thin film systems with intrinsic or induced supramolecular chirality^24,29^. The results are displayed in Figs. 2 and 3 for the F8BT:( +)-2 and F8BT:( −)-2 thin film blends, respectively. Each panel shows (from top to bottom) a microscope image of the dissymmetry parameter *g*_abs_ (detected at 470 nm) including a histogram of the corresponding *g*_abs_ distribution, and also OD_L_, OD_R_ and *g*_abs_ spectra averaged over the entire field of view. For the CD-silent pristine film in panel a of both figures, histograms centred at a *g*_abs_ value of 0 are observed. Likewise, the CD and *g*_abs_ spectra show no CD signal. For the films annealed at 140 °C (panel b), weak CD activity of opposite sign is observed, as indicated by the dominant blue or red colours in the respective CD images. The histograms, which can be well described by a Gaussian distribution, provide *g*_abs_ values of − 0.05 ± 0.08 and 0.03 ± 0.05, respectively. The images show a homogeneous granular structure with granule diameters of 1 μm and below. The slightly negative or positive peaks around 500 nm in the CD and *g*_abs_ spectra confirm the findings obtained from the CD images. Starting from an annealing temperature of 180 °C, the CD images and *g*_abs_ distributions show pronounced changes. Long and extended laminar or filamentary structures start forming, which become particularly pronounced for the films annealed at 220 °C and 260 °C. Consequently, the shape of the *g*_abs_ distributions becomes asymmetric, with an extended tail toward negative or positive *g*_abs_ values for the F8BT:( +)-2 and F8BT:( −)-2 thin film blends, respectively, which need to be fitted by a sum of two or more Gaussian functions, indicating the existence of differently structured chiral regions. Some of the areas exhibit absolute *g*_abs_ values around 1. In the CD and *g*_abs_ spectra, these changes are merely reflected by the size of the CD and *g*_abs_ peaks at 500 nm, without distinct changes in the spectral shape. Such skewed *g*_abs_ distributions are observed for F8BT copolymers with induced chirality for the first time. In our previous investigation, where we employed a methoxy-substituted helicene-like chiral inducer in F8BT films, only Gaussian-shape *g*_abs_ distributions were observed^24^. The structural changes underlying these profound local variations in CD activity are not yet entirely clear. Based on the known phase transitions of F8BT and its blends, one would expect melting into an isotropic liquid crystalline phase at a temperature of roughly 200–210 °C^24,30^, and as shown in our previous work, the addition of such helicene-like chiral inducers changes the transition temperatures of the F8BT polymer only slightly (by less than 1 °C)^24^. Apparently, during the cooling from a hot ($\ge$220 °C) isotropic melt, connected cholesteric blend regions showing large CD activity are formed, in contrast to blends annealed at low temperatures, where the polymer never passes the transition to the isotropic phase. We note that this region of the melt was not accessible in our previous study using the helicene-like additive with two methoxy substituents, as this chiral inducer already displayed some decomposition starting at 200°C^24^. Further experiments using in-situ monitoring by CD microscopy in a temperature-controlled cell will be required to fully understand this effect.

### Broadband Müller matrix spectroscopy

An important open question regarding the supramolecular chiroptical properties of these films is, if the strong CD response contains contributions of “apparent CD” or “pseudo CD”, arising from a combination of linear dichroism and linear birefringence, often summarised under the term LDLB effects^1^. In this respect, broadband Müller matrix spectroscopy is a particularly powerful approach to quantify the full polarisation response of chiral materials^31^. Here, we implemented a new setup covering the wavelength range 420–750 nm which has a polarisation-state generator, consisting of a broadband whitelight LED source, a fixed (0°) linear polariser (P) and a rotatable compensator (C, i.e. a broadband quarter-wave plate) for illuminating the thin film sample (S). Behind the sample, another compensator C’ (rotatable broadband quarter-wave plate) and a fixed (90°) analyser (A, i.e. another linear polariser) served as a polarisation-state detector, resulting in a so-called PCSC’A ellipsometer configuration^32^ with fixed crossed polarisers. The thin films were illuminated at normal incidence, and typically 961 spectra for 31 × 31 angle combinations of the two compensators were recorded using a CCD spectrometer. The spectra were analysed using the linear algebra approach based on the Stokes-Müller calculus developed by Arteaga and co-workers^33^. The measurements provided a set of unnormalised wavelength-dependent Müller matrices, each of which can be conveniently represented in normalised form with the *M*_00_ parameter retained^31^:

1 $${\mathbf{M}} = M_{00} \left( {\begin{array}{*{20}c} 1 & {m_{01} } & {m_{02} } & {m_{03} } \\ {m_{10} } & {m_{11} } & {m_{12} } & {m_{13} } \\ {m_{20} } & {m_{21} } & {m_{22} } & {m_{23} } \\ {m_{30} } & {m_{31} } & {m_{32} } & {m_{33} } \\ \end{array} } \right)$$

*M*_00_ corresponds to the transmitted intensity of the sample for unpolarised light. We note that *m*_03_ and *m*_30_ are roughly related to circular dichroism, *m*_12_ and *m*_21_ are connected with circular birefringence, *m*_01_, *m*_10_, *m*_02_, and *m*_20_ are linked to linear dichroism, and *m*_23_, *m*_32_, *m*_13_, and *m*_31_, are associated with linear birefringence effects^33^. Next, the exact analytical inversion procedure developed by Arteaga and Canillas^34,35^ was applied to the experimental wavelength-dependent Müller matrices **M** to extract the relevant wavelength-dependent optical parameters CD, CB, LD, LD’, LB and LB’ of the thin films, where unprimed quantities are for horizontal and vertical polarization (0° and 90°) and primed quantities refer to the ± 45° reference directions^31,34,36^. In addition, we determined the wavelength-dependent depolarisation parameter $\delta$ which is defined as^36^

2 $$\delta = 1 - \frac{{\sqrt {\left( {\sum\limits_{i,j} {m_{i,j}^{2} } } \right) - m_{0,0}^{2} } }}{{\sqrt 3 \;m_{0,0} }}$$

It assumes a value of 0 in the case of an ideal nondepolarising sample.

**Figure 4:**
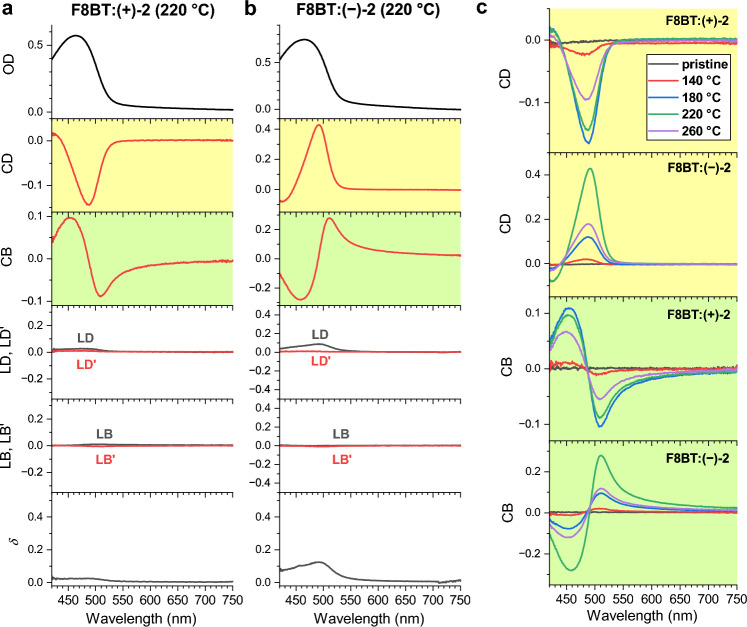
Müller matrix spectra of F8BT:( +)-2 and F8BT:( −)-2 thin films. (**a**) OD, CD (yellow background), CB (green background), LD/LD’ and LB/LB’ response as well as the depolarisation parameter $\delta$ (from top to bottom) for a F8BT:( +)-2 thin film. (**b**) Same as in panel a, but for a F8BT:( −)-2 thin film. (**c**) Temperature-dependent CD and CB spectra for a pristine film (black) and thin films annealed at *T*_ann_ = 140 °C (red), 180 °C (blue), 220 °C (green) and 260 °C (violet), where the upper two panels (yellow background) are for the CD spectra and the lower two panels (green background) are for the CB response of the different thin films.

Panels a and b of Fig. 4 show the results of this analytical inversion of our experimental Müller matrix spectra for the F8BT:( +)-2 and F8BT:( −)-2 thin films annealed at a temperature of 220 °C. Supplementary Figs. 1–10 summarise the Müller matrix spectra for all thin films (pristine and annealed at different temperatures in the range 140–260 °C). In all cases, CD (yellow background) is the dominant contribution to the polarisation response. The CD spectrum of the F8BT:( +)-2 thin film in panel a of Fig. 4 shows peaks at 490 nm (ΔOD =  − 0.144, corresponding to − 4750 mdeg) and 424 nm (ΔOD = 0.079, corresponding to 500 mdeg). In addition, the sample shows a strong CB response (green background) with peaks at 510 nm and 455 nm. The shape of the CD spectrum of the F8BT:( −)-2 thin film panel b of Fig. 4 is practically the mirror image of the F8BT:( +)-2 CD spectrum, with peaks at 492 nm (ΔOD = 0.427, corresponding to 14,100 mdeg) and 425 nm (ΔOD =  − 0.079, corresponding to − 2600 mdeg). This sample also shows a strong CB response with peaks at 510 nm and 458 nm, which resembles the mirror image of the CB spectrum for the F8BT:( +)-2 film. Importantly, the Müller matrix data suggest that contributions from LD/LD’ and LB/LB’ effects average out over the macroscopic length scales detected in this experiment. To prove that this statement also holds for microscopic length scales, one would need to perform Müller matrix microscopy^33,37–39^ which will be carried out in a future investigation. Here, we use a simpler approach: CD microscopy is performed to check for an invariance of the CD images with respect to rotation and flipping of the sample^40,41^. As demonstrated in Supplementary Figs. 11 and 12, the CD response is insensitive to such changes. Therefore, LD/LD’ and LB/LB’ effects also do not play a significant role on micrometre length scales. We note that LD and LD’ effects would be more likely to appear for aligned films, however our spin-coating procedure obviously generates largely unaligned structures (Figs. 2 and 3) consistent with a multidomain cholesteric order featuring a statistical orientation of the individual domains^5,25^. These results are also consistent with previous findings that the CD response of such copolymer–inducer blends is invariant under sample rotation and flipping, also for length scales on the order of the diffraction limit^24^.

The depolarisation parameter $\delta$ is typically close to zero and only in two cases reaches a value of ca. 0.1 near the peak of the CD band (cf. Figure 4b and Supplementary Figs. 9 and 10). Interestingly, this happens for films annealed at 220 °C or 260 °C, which show the aforementioned laminar or filamentary structures of larger size and asymmetric *g*_abs_ distributions (cf. the CD images in panels d and e of Fig. 3). Still, the depolarisation even for these films appears to be weak. Depolarisation is likely caused by light scattering of the micrometre-sized chiral domains of the films.

Panel c of Fig. 4 summarises the dependence of the CD and CB contributions for the F8BT:( +)-2 and F8BT:( −)-2 thin films on the annealing temperature *T*_ann_. No chiral response is observed for the pristine films (black lines). The CD (and also the CB) response of all the annealed films of opposite chirality shows a mirror-image-like shape in the case of F8BT:( +)-2 and F8BT:( −)-2. The peaks of the CD and CB bands increase for *T*_ann_ = 140 °C (red), reach a maximum at annealing temperatures in the range 180–220 °C (blue and green), and decay at 260 °C (violet). Therefore, the optimal temperature range for chiral induction using these helicene-like chiral additives appears to be 180–220 °C. Previous studies for thin films of F8BT and chiral helicene-like inducers with methoxy instead of octyloxy substituents at the dibenzo[*c*,*h*]acridine units showed strong chiral induction already at an annealing temperature of 150°C^24^. Apparently, the chiral inducers with the two long-chain alkoxy groups require an at least 30 K higher annealing temperature to show their optimal chiral induction properties. The decay of the chiral response of the films annealed at 260 °C is likely due to the beginning decomposition of the chiral inducer, as indicated by the slightly reduced absorption peak at 370 nm in Fig. 1 (violet lines in panels b and c). Yet this decomposition occurs at an about 50 °C higher temperature than for the methoxy-analogue investigated previously.

### Transient broadband CD experiments

Ultrafast TrCD spectroscopy in the UV–Vis region was carried out for the different thin films. Here, we implemented an improved version of our previously described TrCD setup^24,29^, which originally used a linearly polarised pump laser beam. According to Kliger, Simon and co-workers, time-resolved absorptive CD experiments can be plagued by several effects^42^: First of all, LD intrinsic to a sample can couple with strain birefringence (LB') in the optics before the sample (such as a glass substrate) and distort the TrCD response. However, because our thin films have negligible LD, as shown by the Müller matrix spectra in Fig. 4a and b and Supplementary Figs. 1–10, we expect this effect to be small. Next, LD effects induced by a linearly polarised pump beam need to be considered, because they can induce a transient anisotropy by photoselection^42^. Such pump-induced LD can couple with stray LB of the optics before the sample, and transient contributions arising from this effect may overlap with the TrCD signal^42^. Indeed, this could be the reason why some of our previously recorded TrCD spectra employing a pump beam with linear polarisation for thin-film samples of opposite chirality did not resemble a perfect mirror symmetry^24^, despite the fact that they were carried out at the “magic angle” condition for TrCD spectroscopy^43–45^.

**Figure 5:**
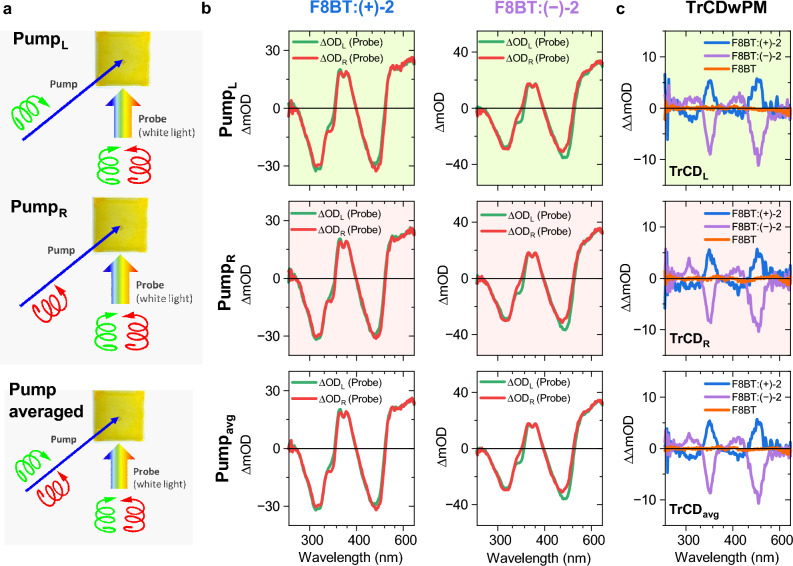
TrCD spectroscopy with Pump Modulation (TrCDwPM) for chiral F8BT:( +)-2 and F8BT:( −)-2 thin film blends annealed at 220 °C as well as an achiral F8BT thin film. (**a**) Illustration of the TrCD experiment with polarisation modulation of the pump beam: LCP pump beam (top) and RCP pump beam (middle) providing access to an experiment with randomly polarised excitation (bottom) due to the noncoherent nature of the two pump processes. In each case, the photoinduced dynamics is alternately detected by LCP and RCP supercontinuum probe pulses. (**b**) Corresponding transient absorption spectra of F8BT:( +)-2 (left column) and F8BT:( −)-2 (right column) for excitation with an LCP pump beam (top row) or an RCP pump beam (middle row) providing the pump-polarisation-averaged spectra (bottom row). Transient absorption spectra for an LCP probe beam (ΔOD_L_, green solid lines) and an RCP probe beam (ΔOD_R_, red solid lines) are shown in each case. (**c**) Resulting TrCD spectra (ΔOD_L_ − ΔOD_R_) for LCP excitation (TrCD_L_, top), for RCP excitation (TrCD_R_, middle) and the pump-polarisation-averaged spectra (TrCD_avg_ = (TrCD_L_ + TrCD_R_)/2). Results for F8BT:( +)-2 (blue solid lines) and F8BT:( −)-2 (violet solid lines) thin films as well as an achiral F8BT thin film without chiral additive (orange solid lines) are shown. In all cases, the samples were excited at 400 nm, and 21 spectra in the time-delay range 1–3 ps were averaged.

To minimise such unwanted pump-induced effects, an approach using randomly polarised excitation was implemented, as illustrated in Fig. 5a: Here, we performed TrCD experiments with alternating LCP and RCP pump pulses at 400 nm, denoted as TrCD_L_ and TrCD_R_, respectively. We averaged these two TrCD measurements (TrCD_avg_ = (TrCD_L_ + TrCD_R_)/2), and because the two individual TrCD spectra are for an excitation by two separate, noncoherent LCP and RCP pump pulses, TrCD_avg_ is equivalent to a TrCD spectrum obtained for randomly polarised excitation^46^. This strategy, denoted as “TrCDwPM” (TrCD with Pump Modulation), largely eliminates the aforementioned problems regarding photoselection occurring for a linearly polarised pump pulse. Residual, weak elliptical polarisation may still be present, because the pump and probe beams do not propagate in an ideally collinear fashion due to geometric restrictions of the optical setup.

The effectiveness of this approach is demonstrated in panels b and c of Fig. 5, which display transient absorption and TrCD spectra for chiral F8BT:( +)-2 and F8BT:( −)-2 thin films (annealed at 220 °C) as well as an achiral F8BT thin film (without chiral inducer), averaged over the time interval 1–3 ps. Starting with the transient absorption spectra in the top row of panel b (excitation by an LCP pump pulse at 400 nm), we observe two ground-state bleach (GSB) bands at 465 and 320 nm, corresponding to the S_0_ → S_1_ transition and higher S_0_ → S_n_ transitions of F8BT, respectively. In addition, pronounced S_1_ → S_n_ excited-state absorption (ESA) bands of F8BT are observed at 670 and 370 nm. The small dip at 368 nm arises from a weak, overlapping GSB signal of the chiral inducer. The transient absorption spectra obtained for probing with LCP and RCP pulses (ΔOD_L_ and ΔOD_R_, displayed as green and red lines, respectively) show distinct differences, which are most pronounced around 490 and 345 nm, where peaks of the steady-state CD spectra are located (Fig. 1d and e). The spectra in the second row for excitation by an RCP pump pulse at 400 nm essentially show the same difference, as do the averaged spectra for LCP and RCP excitation in the third row.

Note that for the two chiral thin films of opposite chirality, ΔOD_L_ and ΔOD_R_ are essentially “switched”, meaning that the resulting TrCD spectra (ΔOD_L_ − ΔOD_R_) should look like mirror images. This is indeed the case, as demonstrated in panel c of Fig. 5, which (from top to bottom) shows TrCD_L_, TrCD_R_ and the pump-averaged spectrum TrCD_avg_, where the blue and violet solid lines are for the F8BT:( +)-2 and F8BT:( −)-2 thin films, respectively. Each of these TrCD signals resembles the inverted steady-state CD spectrum of the respective film (Fig. 1d and e), and so there are no clear indications for excited-state CD bands. The about a factor of two larger TrCD signal for the F8BT:( +)-2 thin film can be traced back to its already larger steady-state CD response (see Fig. 1). For comparison, we also included the TrCD spectra for an achiral F8BT film without chiral inducer (orange solid lines), which shows no TrCD signal.

**Figure 6:**
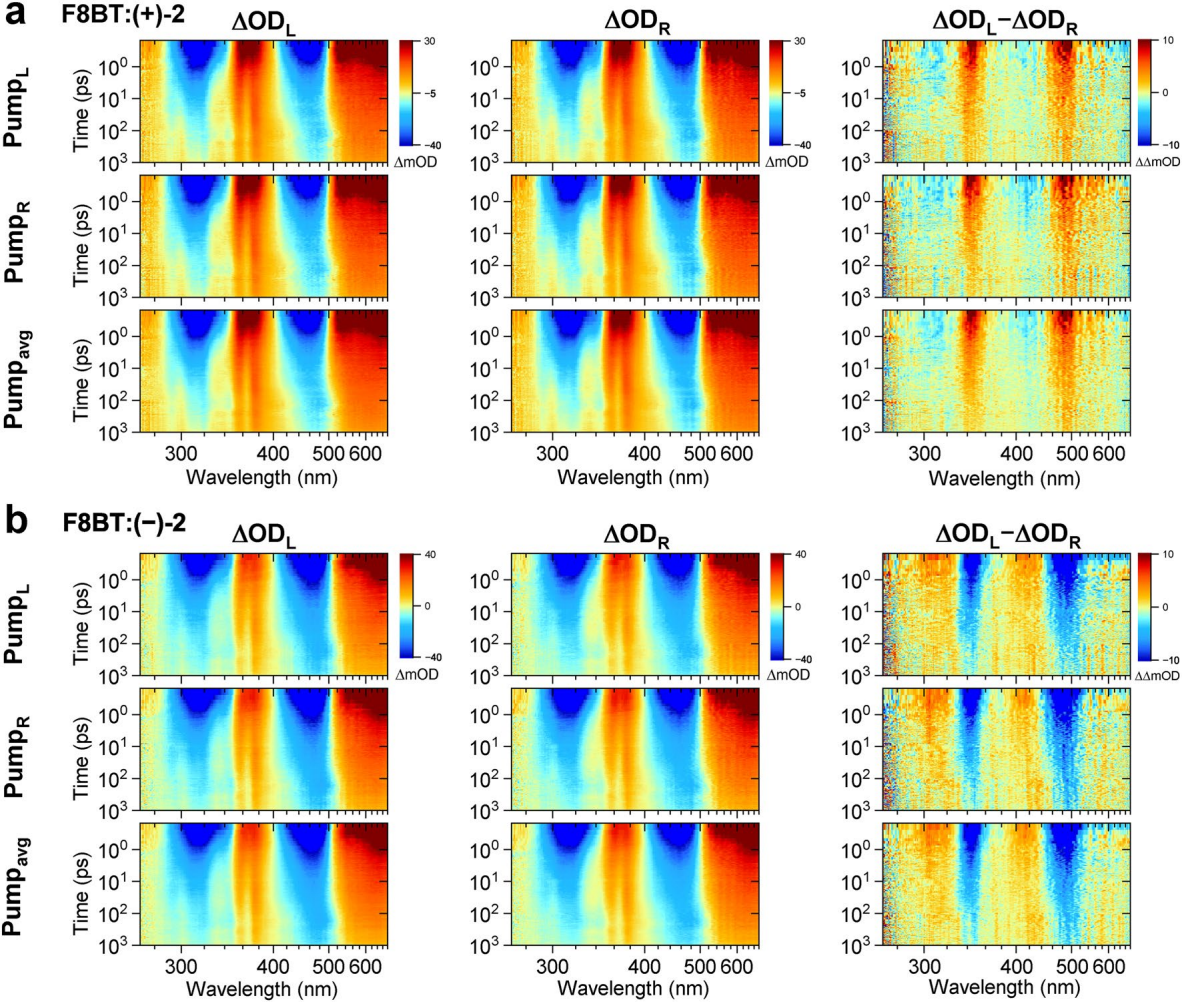
Contour plots of transient spectra with pump modulation for chiral F8BT:( +)-2 and F8BT:( −)-2 thin film blends annealed at 220 °C. (**a**) Transient spectra of the F8BT:( +)-2 blend for excitation by an LCP pump pulse (first row) or an RCP pump pulse (second row), as well as for the average of LCP and RCP excitation representing random polarisation (third row). The first two columns show the transient absorption spectra probed by an LCP supercontinuum (ΔOD_L_) and an RCP supercontinuum (ΔOD_R_), respectively, and the third column displays the TrCD spectrum, which corresponds to the difference ΔOD_L_ − ΔOD_R_. (**b**) Same as in panel a, but for the F8BT:( −)-2 blend. Pump wavelength: 400 nm. Note the logarithmic time axes.

While Fig. 5 compared the spectra at early times only, in Fig. 6 the complete spectral evolution for F8BT:( +)-2 (panel a) and F8BT:( −)-2 (panel b) is summarised as a contour plot. In addition, Supplementary Fig. 13 shows selected transient spectra for these two systems at pump–probe delay times of 1, 2, 10, 100 and 980 ps. The GSB bands at 465 and 320 nm in the transient absorption spectra (first and second column) and likewise the “CD bleach” bands at 490 and 345 nm in the TrCD spectra (third column) have already decayed to about 50% of their initial amplitude by 10 ps, and this decay subsequently slows down considerably. The fast initial decay, which is much faster than the intrinsic lifetime of the F8BT copolymer in neat thin films (about 240 ps)^24^, arises from diffusive singlet–singlet annihilation (SSA, S_1_ + S_1_ → S_n_ + S_0_, where S_n_ is a higher excited singlet state). The SSA process dominates at the initial S_1_ exciton number density of about 10^19^ cm^−3^ employed in these time-resolved experiments. Its bimolecular rate constant is about 1 × 10^−7^ cm^3^ s^−1^, and this initial fast decay and the subsequent slowing-down are characteristic for such a second-order annihilation process^24^. As mentioned above, the TrCD signal does not show clear excited-state CD contributions and thus mainly reflects the recovery of S_0_ population from S_1_. It is therefore ideally suited to exclusively monitor the recovery of the electronic ground state of the polymer, which allows for the aforementioned direct and easy determination of the rate constant for the SSA process. In contrast, such a kinetic modelling is more difficult in transient absorption experiments, because there might be an overlap of bands from other electronic species. For instance, the long-lived residual absorption signal above 500 nm, which differs from the S_1_ ESA signal at early times (compare the spectra at 1 and 980 ps in Supplementary Fig. 13) indicates the formation of a smaller fraction (< 30%) of a long-lived charge-pair state^24,29,47^. This charge-pair state is generated from the high-energy S_n_ state, which is populated via the aforementioned SSA process.

**Figure 7:**
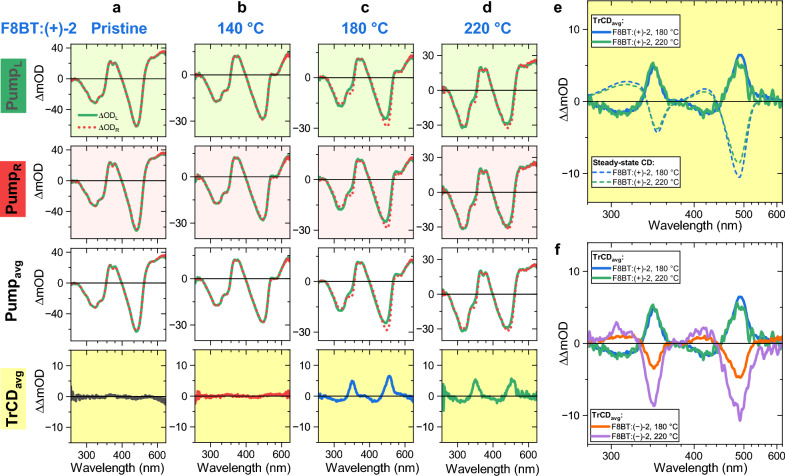
TrCD spectra with pump modulation of F8BT:( +)-2 and F8BT:( −)-2 thin films in the pristine state and annealed at different temperatures. Transient absorption spectra of a pristine F8BT:( +)-2 thin film (**a**) and the F8BT:( +)-2 thin films annealed at 140 °C (**b**), 180 °C (**c**) and 220 °C (**d**), for excitation by an LCP pump beam (first row) or an RCP pump beam (second row) leading to the pump-polarisation-averaged spectra (third row, i.e. (Pump_L_ + Pump_R_)/2). In each case, the transient absorption spectra for an LCP probe beam (ΔOD_L_, green solid lines) and an RCP probe beam (ΔOD_R_, red dotted lines) are shown. The bottom row (yellow background) shows the resulting pump-polarisation-averaged spectra TrCD_avg_ (ΔOD_L_ − ΔOD_R_). (**e**) Comparison of the pump-polarisation-averaged TrCD spectra at 180 and 220 °C of F8BT:( +)-2 with the corresponding steady-state CD spectra of F8BT:( +)-2. (**f**) Comparison of the pump-polarisation-averaged TrCD spectra at 180 and 220 °C of the F8BT:( +)-2 and F8BT:( −)-2 thin films. Pump wavelength: 400 nm. 21 spectra in the time-delay range 1–3 ps were averaged in each case.

Figure 7 highlights the impact of the different annealing conditions on the TrCD response. In panels a and b, essentially no differences between the transient absorption spectra ΔOD_L_ and ΔOD_R_ (for LCP and RCP probing, respectively) are found for the pristine film and the weakly CD-active F8BT:( +)-2 film annealed at 140 °C, and therefore practically no TrCD signal is observed (bottom panels with yellow background). This finding is independent of the fact if the films were initially excited by LCP or RCP pump pulses. In contrast, the F8BT:( +)-2 films annealed at 180 and 220 °C show pronounced differences between ΔOD_L_ and ΔOD_R_, as discussed for Fig. 5, and thus substantial pump-averaged TrCD signals of the same sign and similar amplitude are observed for these two films. Note that the amplitudes of the transient absorption signals of these films are comparable with those of the CD-silent pristine F8BT layer. This clearly indicates that the transition to the chiral nematic phase is a necessary condition to obtain a TrCD signal. The chiral supramolecular order is therefore a prerequisite for a substantial TrCD signal in these cholesteric copolymer systems.

Panel e compares the TrCD_avg_ spectra for the F8BT:( +)-2 films annealed at 180 and 220 °C with the corresponding steady-state CD spectra of the same two films (see Fig. 1d). In both cases, the TrCD spectra resemble mirror images of the steady-state spectra without noticeable contributions from excited-state CD activity. This can be understood based on the fact that the electronically excited F8BT chromophores in the film are spaced quite far apart from each other, because the fraction of F8BT units excited by the pump pulse is on the order of 10%^29^. Therefore, coupling between the S_1_ chromophores within one chiral nematic layer and across these layers is probably too weak to result in a sizeable supramolecular transient excited-state CD signal. The observed TrCD_avg_ signal is thus essentially a “bleached” CD signal, because of the “missing” S_0_ chromophores which have been promoted to the S_1_ state.

Panel f shows a comparison of the F8BT:( +)-2 films annealed at 180 and 220 °C with their F8BT:( −)-2 counterparts annealed at the same temperatures. As a result of the opposite helical sense of the supramolecular cholesteric structures, the shape of the TrCD spectra show mirror symmetry, and thus chiral discrimination on ultrafast time scales is achieved for these systems. Note that the TrCD amplitudes also nicely mirror the differences observed in the steady-state CD spectra of Fig. 1 (panels d and e).

In conclusion, we have introduced a new helicene-like chiral additive which is able to induce strong supramolecular chirality in the polyfluorene-based copolymer F8BT. The chiral additive has very good thermal stability, which allows an extended range of annealing temperatures to be explored up to the isotropic phase of the F8BT–inducer blends. Measurements on a new setup for broadband Müller matrix spectroscopy identified the main contributions to the chiroptical response of these films, namely CD and CB. The results from ultrafast CD spectroscopy demonstrated that averaging experiments for excitation by ultrashort, randomly polarised LCP and RCP light pulses provides a clean transient CD response, including an almost perfect mirror symmetry of the spectra for films of opposite chirality. This so-called TrCDwPM method is therefore the method of choice for follow-up studies of CD-active systems. In the future it will be also interesting to address the emission properties of these films in detail, specifically the CPL response as a function of the annealing temperature and compare the resulting dissymmetry parameters *g*_lum_ with the *g*_abs_ values determined here.

## Methods

### Synthesis of compounds ( +)-2 and ( −)-2

In a sealable glass tube, a solution of the ( +)-bisphenol precursor^26^ (602 mg, 0.80 mmol) and cesium carbonate (1.55 g, 4.76 mmol, 6 eq) in 12 mL DMF was stirred for 10 min at room temperature before addition of 1-bromooctane (1.00 g, 5.18 mmol, 6.5 eq). The tube was sealed and the reaction mixture heated for 2 days at 80 °C. After evaporation, the crude was placed in water (15 mL) and the product was extracted with CH_2_Cl_2_ (20 mL). The organic layer was washed three times with water, dried over sodium sulfate and evaporated. After purification by SiO_2_ column chromatography using a gradient of pentane/CH_2_Cl_2_ 1/0 to 1/3 as the eluent, compound ( +)-2 (421 mg, 65%) was obtained as a brown solid: ^**1**^**H NMR** (400.14 MHz, CDCl_3_): *δ* = 0.87 (6H, t, *J* = 7.19 Hz), 1.0–1.4 (24H, m), 2.31 (2H, m), 2.76 (2H, m), 2.91 (4H, t, *J* = 7.57 Hz), 3.58 (4H, td, *J* = 9.05 Hz, *J* = 6.80 Hz), 3.77 (4H, td, *J* = 9.05 Hz, *J* = 6.80 Hz), 6.94 (2H, d, *J* = 8.20 Hz), 7.20 (2H, d, *J* = 8.16 Hz), 7.32 (2H, td, *J* = 8.12 Hz, *J* = 1.15 Hz), 7.47 (2H, d, *J* = 8.47 Hz), 7.50 (2H, td, *J* = 8.02 Hz, *J* = 1.15 Hz), 7.60 (4H, d, *J* = 9.68 Hz), 7.71 (2H, d, *J* = 7.84 Hz), 7.87 (2H, d, *J* = 8.20 Hz). ^**13**^**C NMR** (100.62 MHz, CDCl_3_): *δ* = 14.33 (CH_3_), 22.68 (CH), 25.71 (CH), 29.21 (CH), 29.26 (CH), 29.46 (CH), 29.50 (CH), 31.79 (CH), 69.32 (CH), 114.29 (CH), 124.45 (Cq), 124.74 (CH), 125.64 (CH), 125.99 (CH), 126.12 (CH), 126.42 (CH), 126.79 (CH), 126.95 (CH), 129.94 (CH), 132.13 (Cq), 132.30 (CH), 132.44 (Cq), 132.79 (Cq), 132.96 (Cq), 134.41 (Cq), 144.12 (Cq), 153.24 (Cq), 156.10 (Cq). **HMRS (ESI)** [M + H]^+^: calculated for C_58_H_61_N_2_O_2_ 817.4722, found 817.4728. The ^1^H NMR and ^13^C NMR spectra as well as the ESI mass spectrum can be found in Supplementary Figs. 14–16.

The same procedure was used for the synthesis of compound ( −)-2, starting from the ( −)- bisphenol precursor^26^ (370 mg, 0.49 mmol), and gave compound ( −)-2 (205 mg, 51%), which was obtained as a brown viscous oil.

### Preparation and characterisation of thin films

Borosilicate glass slides (Duran Wheaton Kimble, thickness 1 mm) were used as substrates for the thin films. The substrates were thoroughly cleaned and then treated by UV-C radiation for 15 min. F8BT (Merck, *M*_*n*_ ≤ 25,000 g mol^−1^) was used as received. F8BT and one of the enantiopure helicene-like compounds ( +)-2 or ( −)-2 were dissolved in a mixture of chloroform and chlorobenzene (both from Merck, > 99% and ≥ 99.8%, respectively) in a ratio of 9:1 (v:v). The concentrations of F8BT and the chiral additive were 7.50 mg mL^−1^ and 3.75 mg mL^−1^, respectively (33 wt% of the chiral additive). The solution was sonicated for 60 min, filtered (PTFE filter, pore size 0.45 µm) and then spin-coated onto the substrates at 500 rpm for 60 s in a nitrogen-filled glovebox (humidity: < 1%). Subsequently, the films were either left in their pristine state or annealed on a hot plate for 15 min at temperatures between 140 and 260 °C. The method of picosecond ultrasonics^48,49^ was employed to determine the thickness *d* of selected films as described previously^50^. The thickness was obtained as *d* = 0.25·$\tau$_a_·*c*_L_. Here, $\tau$_a_ is the coherent acoustic phonon oscillation period obtained from the transient absorption kinetics, which were averaged over the wavelength range 490–520 nm, and *c*_L_ is the known longitudinal sound velocity of benzo[2,1,3]thiadiazol-based copolymers (2490 m s^−1^)^50^. Representative results from picosecond ultrasonics for the current films can be found in Supplementary Fig. 17. In addition, the thickness dependence of the dissymmetry parameter *g*_abs_ was investigated, and representative results for F8BT:( −)-2 thin films are provided in Supplementary Fig. 18. Here, a systematic variation of the film thickness was achieved either by different rotational speeds, dilution of the solution used for spin-coating (to arrive at thinner layers) or by introducing an additional loading time of about 30 s prior to the spin-coating process (to obtain thicker layers).

### Steady-state absorption and circular dichroism spectroscopy and microscopy

Steady-state absorption spectra of the thin films were recorded on a Varian Cary 5000 spectrophotometer with a slit width of 0.5 nm. CD spectra were measured on the same instrument with the aid of a home-built add-on reported previously^29^ which employed two interchangeable combinations of a polariser and an achromatic quarter-wave plate, which seamlessly cover the UV–vis range. CD microscopy images were measured on a modified version of an inverted microscope setup based on an Olympus IX71 frame, as described previously^29^. Instead of the manual rotation mount used previously, this setup now employs a rotation mount with resonant piezoelectric motors (Thorlabs ELL14K) in the illumination path, which rotates the fast axis of the quarter-wave plate by + 45° or − 45° with respect to the polariser axis with a bidirectional accuracy of better than 0.4°.

### Broadband Müller matrix spectroscopy

The complete polarisation response of the thin films was measured using a newly implemented setup for Müller matrix spectroscopy which in its current configuration can measure spectra over the wavelength range 420–750 nm. The broadband emission from a continuous-wave whitelight LED (Thorlabs MNWHL4) was collimated by an aspheric condenser lens (Thorlabs ACL2520U-A) and sent through a linear film polariser (Thorlabs LPVISE100-A) set at vertical polarisation (0°). The beam then passed through an achromatic quarter-wave plate (Thorlabs AQWP05M-580) which was held in a rotation mount with resonant piezoelectric motors (Thorlabs ELL14K). After passing the thin film sample, which was mounted at normal incidence, the beam traversed another achromatic quarter-wave plate (Thorlabs AQWP05M-580), which was attached to another piezo-driven rotation mount (Thorlabs ELL14K). The light then passed through another linear film polariser (Thorlabs LPVISE100-A) set at horizontal polarisation (90°) and was focused by a quartz lens into a fibre-optic quartz cable which was connected to a spectrograph with a back-illuminated thermoelectrically cooled CCD detector (Avantes AvaSpec-Hero). Measurements were typically performed by moving both quarter-wave plates between + 100° to –100° in 31 steps, resulting in a set of spectra for a total of 961 angle combinations. These spectra were evaluated using the linear algebraic approach based on the Stokes-Müller calculus as described by Arteaga and co-workers^33^. Subsequently, the exact analytical inversion procedure developed by Arteaga and Canillas^34,35^ was applied to the set of experimental Müller matrix spectra to extract the wavelength-dependent CD, CB, LD, LD’, LB and LB’ response of the chiral thin film blends. Imperfections of the polarisation optics were accounted for by measuring a separate Müller matrix for an uncoated borosilicate glass substrate.

### Ultrafast broadband TrCD spectroscopy with pump pulse modulation

The experimental setup for TrCD spectroscopy was described in detail previously^24,29^ and only the experimental changes relevant to the current measurements are described here. The thin-film samples were excited by the second harmonic (400 nm) of the amplified titanium:sapphire laser system. Switching between left and right circular polarisation of this pump beam was achieved by an achromatic quarter-wave plate (Thorlabs AQWP05M-580). It was placed in a computer-controlled rotation mount with resonant piezoelectric motors (Thorlabs ELL14K), which rotated the fast axis of the quarter-wave plate by + 45° or − 45° with respect to the polarisation axis of the pump beam. The resulting transient circular dichroism signals were obtained as ΔΔOD_Pump,L_ = (ΔOD_Probe,L_ − ΔOD_Probe,R_)_Pump,L_ and ΔΔOD_Pump,R_ = (ΔOD_Probe,L_ − ΔOD_Probe,R_)_Pump,R_, for LCP and RCP pump, respectively. Here, each of the ΔΔOD measurements was obtained from four consecutive laser shots (LCP and RCP supercontinuum probe, each with and without the pump beam). The measurements of ΔΔOD_Pump,L_ and ΔΔOD_Pump,R_ (1000 laser shots each) for different pump–probe delay times Δ*t*_*i*_ were measured in the sequence ΔΔOD_Pump,L_(Δ*t*_1_) → ΔΔOD_Pump,R_(Δ*t*_1_) → ΔΔOD_Pump,R_(Δ*t*_2_) → ΔΔOD_Pump,L_(Δ*t*_2_) → … to minimise the wear-out of the piezoelectric motors in the rotation mount. Three of these runs were averaged to provide the final signal, resulting in a total of 3000 laser shots. The transient circular dichroism signal ΔΔOD_avg_ for randomly polarised excitation was then obtained as (ΔΔOD_Pump,L_ + ΔΔOD_Pump,R_)/2.

### Supplementary Information

Supplementary Information.

## Data Availability

The authors declare that the data supporting the findings of this study are available within the paper and its supplementary information files.
