# Supplementary material for: Annealing temperature-dependent induced supramolecular chiroptical response of copolymer thin films studied by pump-modulated transient circular dichroism spectroscopy
Source: Sci Rep. 2024 Jun 3;14:12694. doi: 10.1038/s41598-024-63126-4 (PMC11148131; doi:10.1038/s41598-024-63126-4)
Supplement: Supplementary file 1 — Supplementary Information. [file 41598_2024_63126_MOESM1_ESM.pdf]

Supplementary Information for

Annealing temperature-dependent induced supramolecular chiroptical response of copolymer thin films studied by pump-modulated transient circular dichroism spectroscopy

Domenic Gust[1], Mirko Scholz[1], Vivien Schumacher[1], Jean-Christophe Mulatier[2], Delphine Pitrat[2], Laure Guy[2], Kawon Oum[1*] & Thomas Lenzer[1*]

[1] University of Siegen, Faculty IV: School of Science and Technology, Department Chemistry and Biology, Physical Chemistry 2, Adolf-Reichwein-Str. 2, 57068 Siegen, Germany
E-mail: oum@chemie.uni-siegen.de, lenzer@chemie.uni-siegen.de

[2] Univ. Lyon, ENS de Lyon, CNRS UMR 5182, Université Claude Bernard Lyon 1, Laboratoire de Chimie, F69342 Lyon, France

Inventory of Supplementary Information

Supplementary Note 1. Müller matrix spectra of F8BT:(+)-2 and F8BT:(−)-2 thin films S2

Supplementary Note 2. Invariance of CD images under sample rotation and flipping S12

Supplementary Note 3. Additional transient absorption and TrCD spectra of thin films........S14

Supplementary Note 4. NMR and mass spectra S15

Supplementary Note 5. Coherent acoustic phonon dynamics of thin films S18

Supplementary Note 6. Thickness dependence of g_{abs} for F8BT:(−)-2 thin films....................S19

Supplementary Note 1.
Müller matrix spectra of F8BT:(+)-2 and F8BT:(−)-2 thin films

Supplementary Figs. 1–10 summarise the results from Müller matrix spectroscopy for a range of pristine and annealed F8BT:(+)-2 and F8BT:(−)-2 thin films.

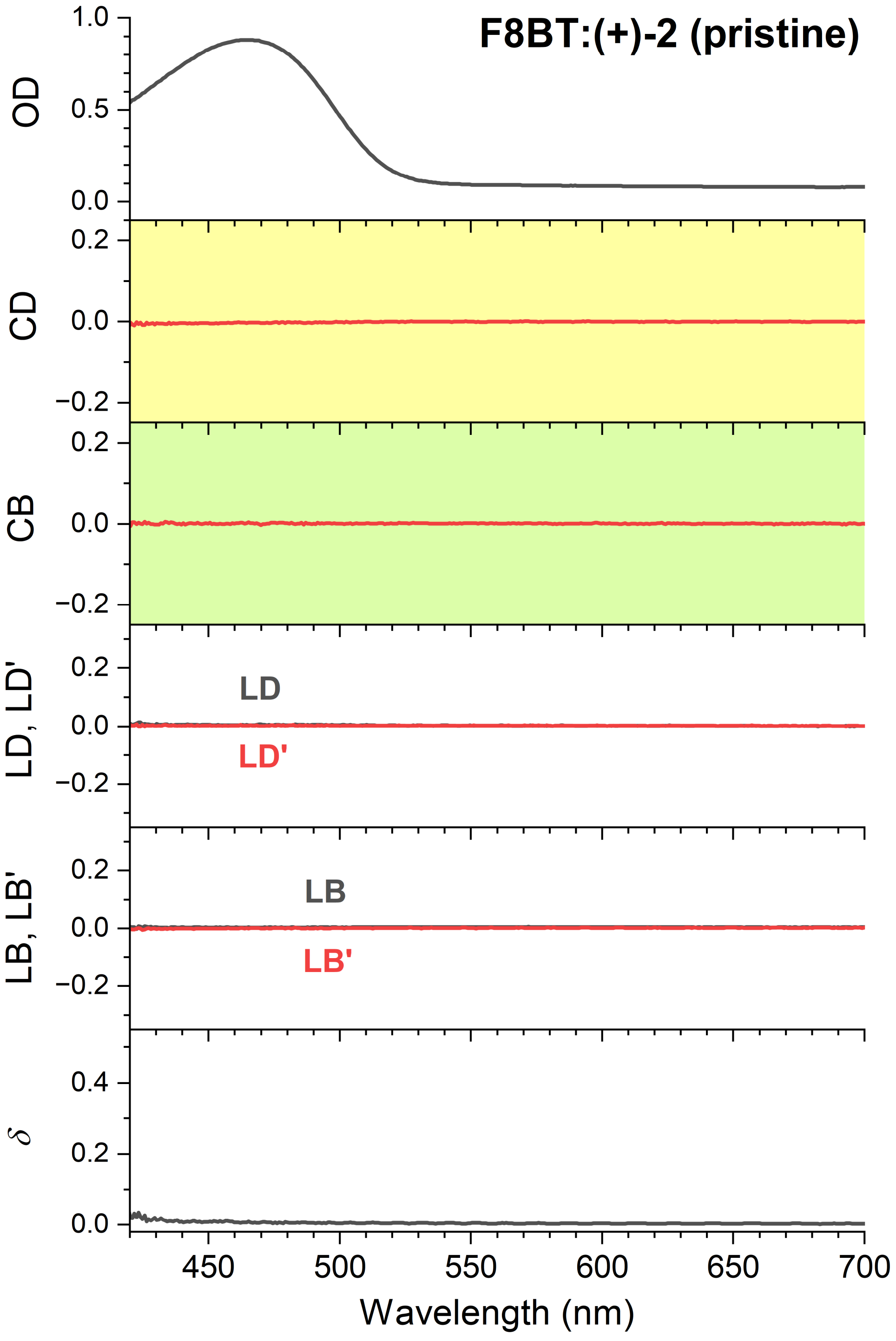

Supplementary Figure 1. Müller matrix spectra of a pristine F8BT:(+)-2 thin film. OD, CD (yellow background), CB (green background), LD/LD′ and LB/LB′ response as well as the depolarisation parameter δ (from top to bottom).

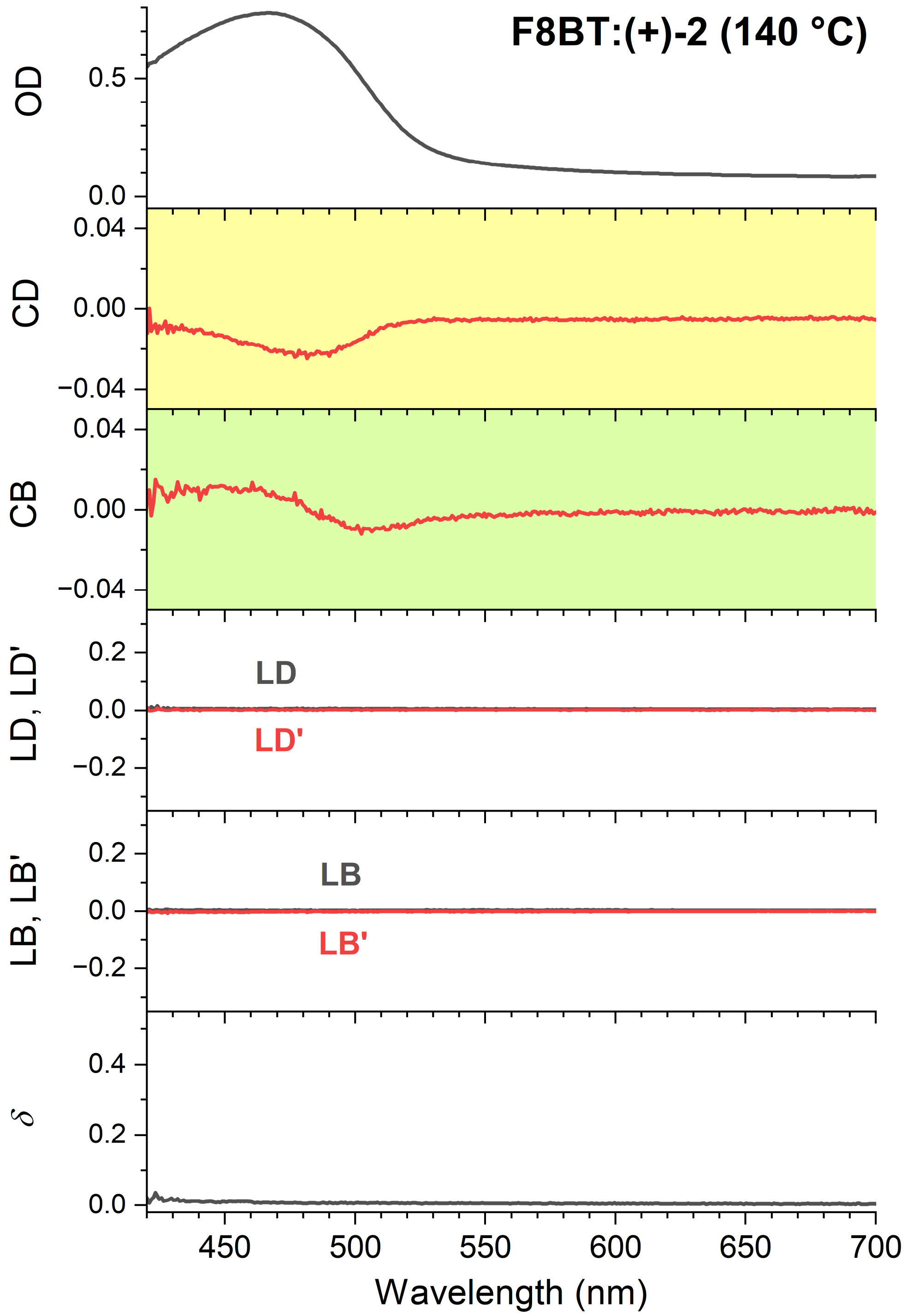

Supplementary Figure 2. Müller matrix spectra of an F8BT:(+)-2 thin film annealed at 140 °C. OD, CD (yellow background), CB (green background), LD/LD′ and LB/LB′ response as well as the depolarisation parameter δ (from top to bottom).

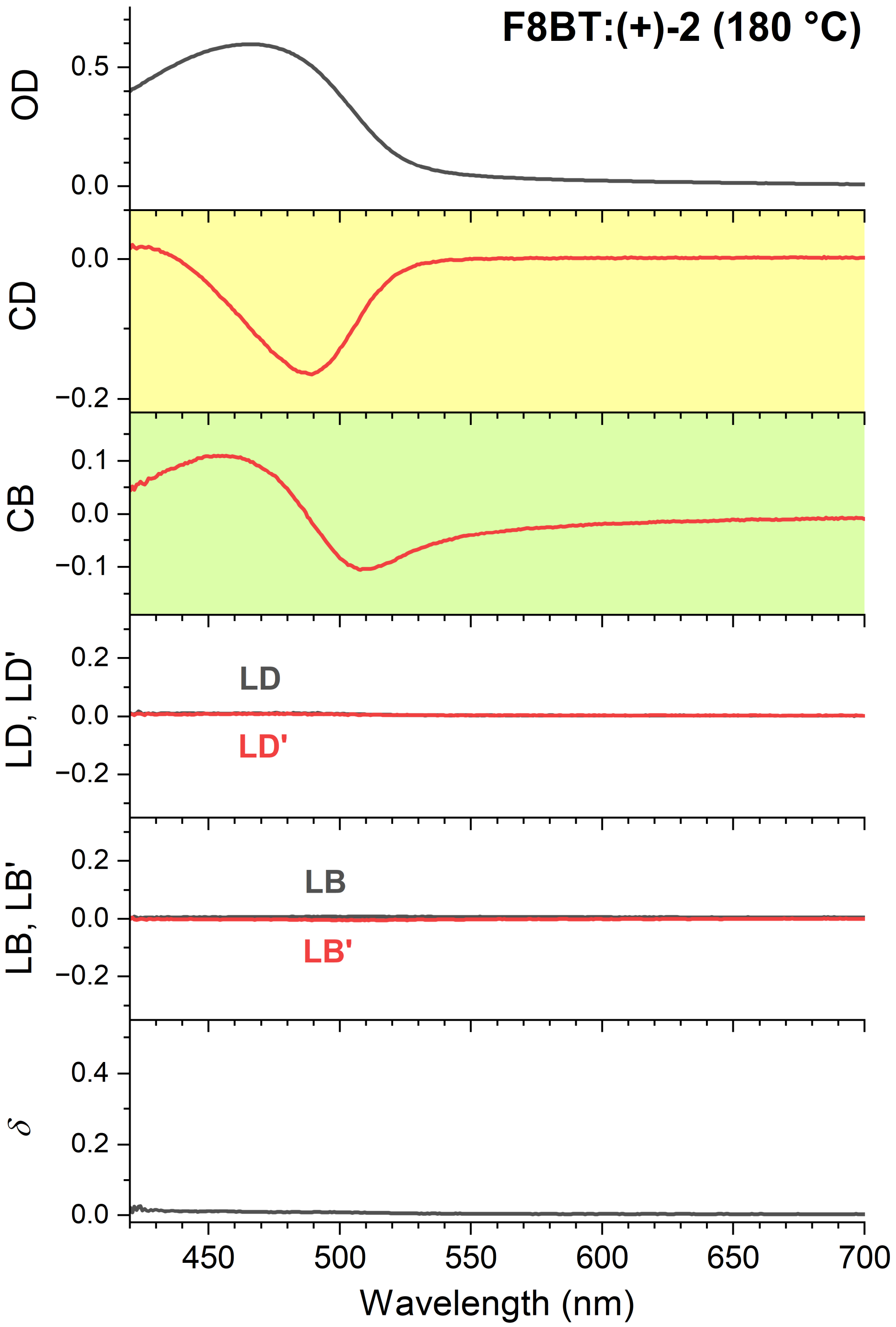

Supplementary Figure 3. Müller matrix spectra of an F8BT:(+)-2 thin film annealed at 180 °C. OD, CD (yellow background), CB (green background), LD/LD’ and LB/LB’ response as well as the depolarisation parameter δ (from top to bottom).

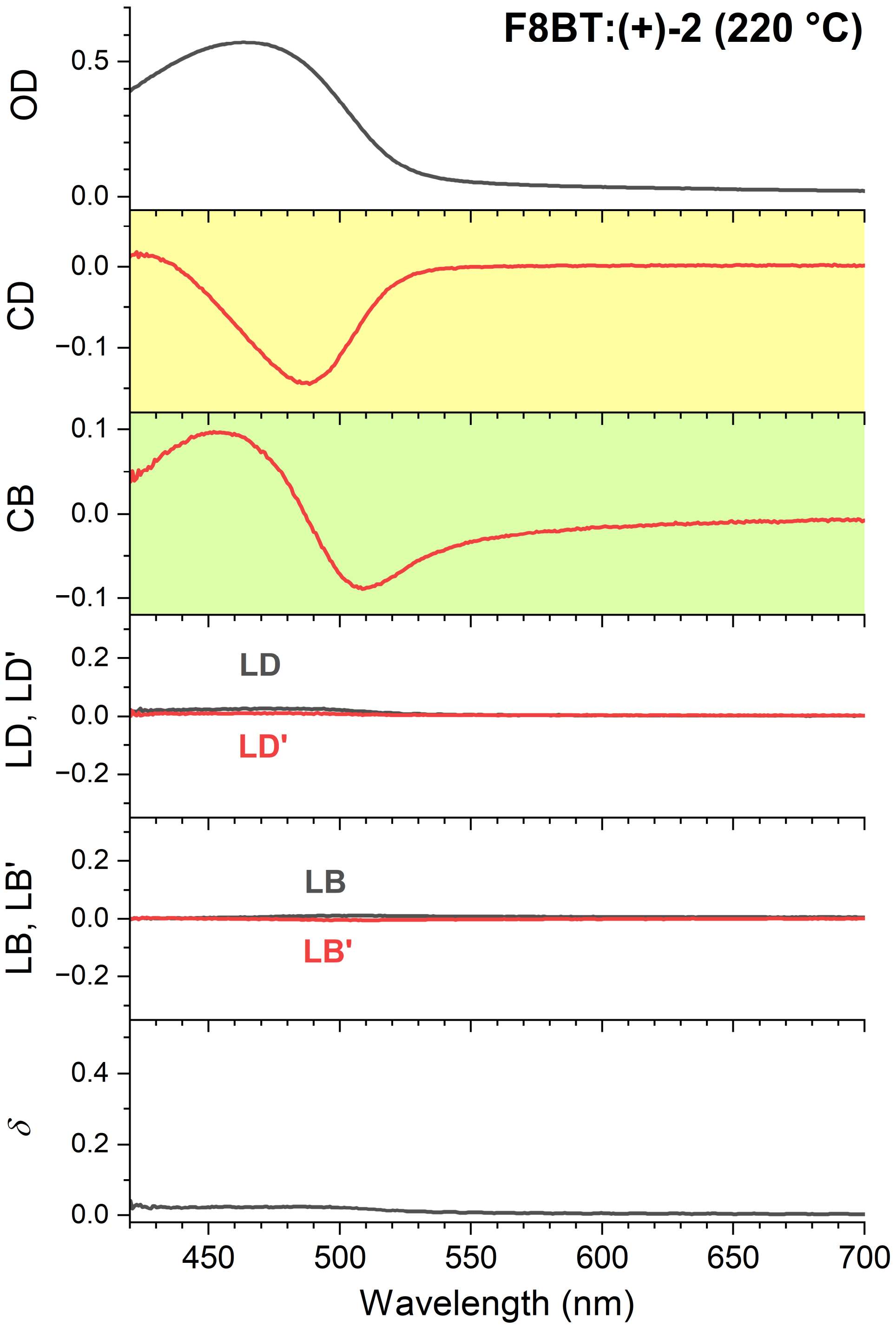

Supplementary Figure 4. Müller matrix spectra of an F8BT:(+)-2 thin film annealed at 220 °C. OD, CD (yellow background), CB (green background), LD/LD' and LB/LB' response as well as the depolarisation parameter δ (from top to bottom).

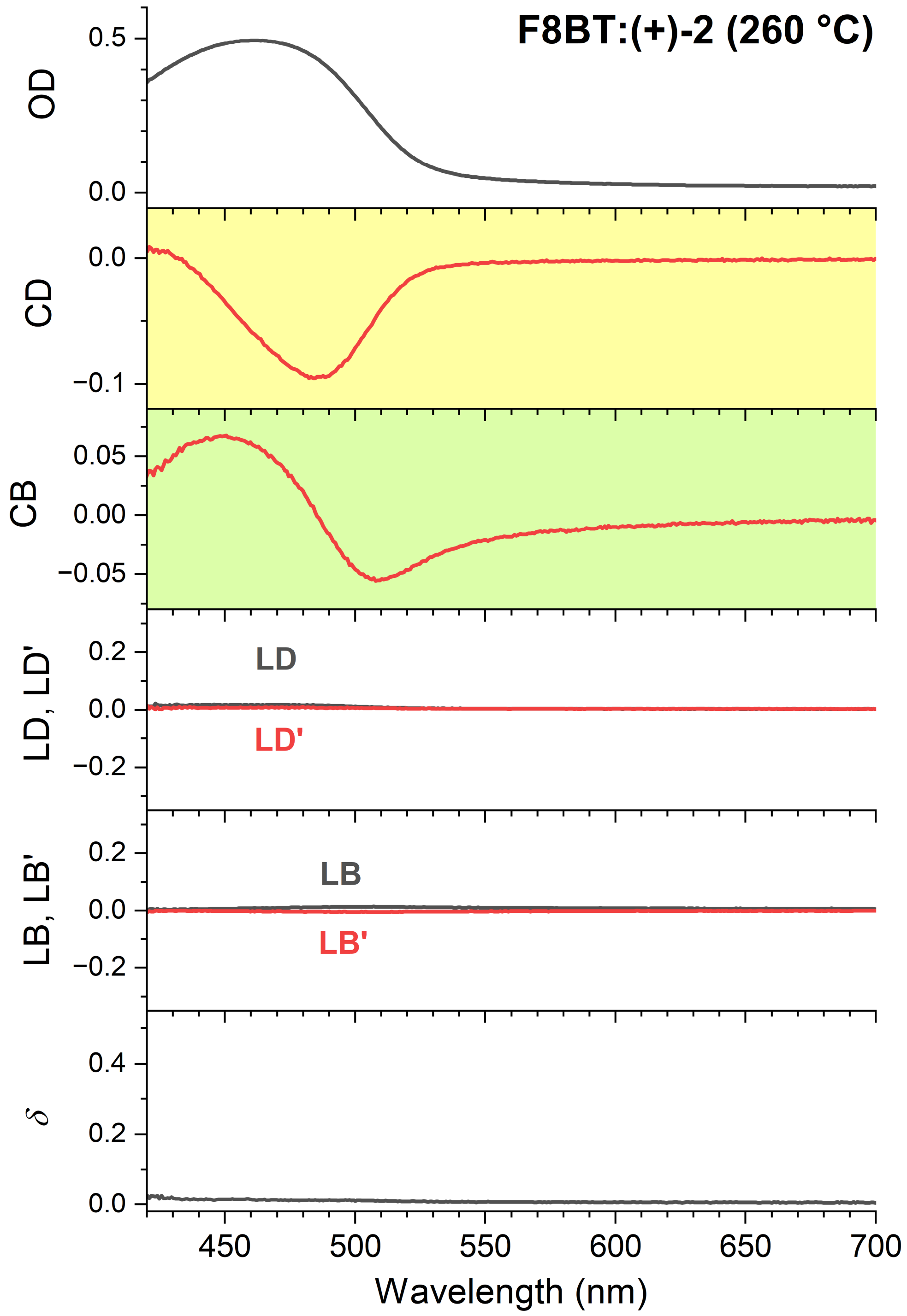

Supplementary Figure 5. Müller matrix spectra of an F8BT:(+)-2 thin film annealed at 260 °C. OD, CD (yellow background), CB (green background), LD/LD' and LB/LB' response as well as the depolarisation parameter δ (from top to bottom).

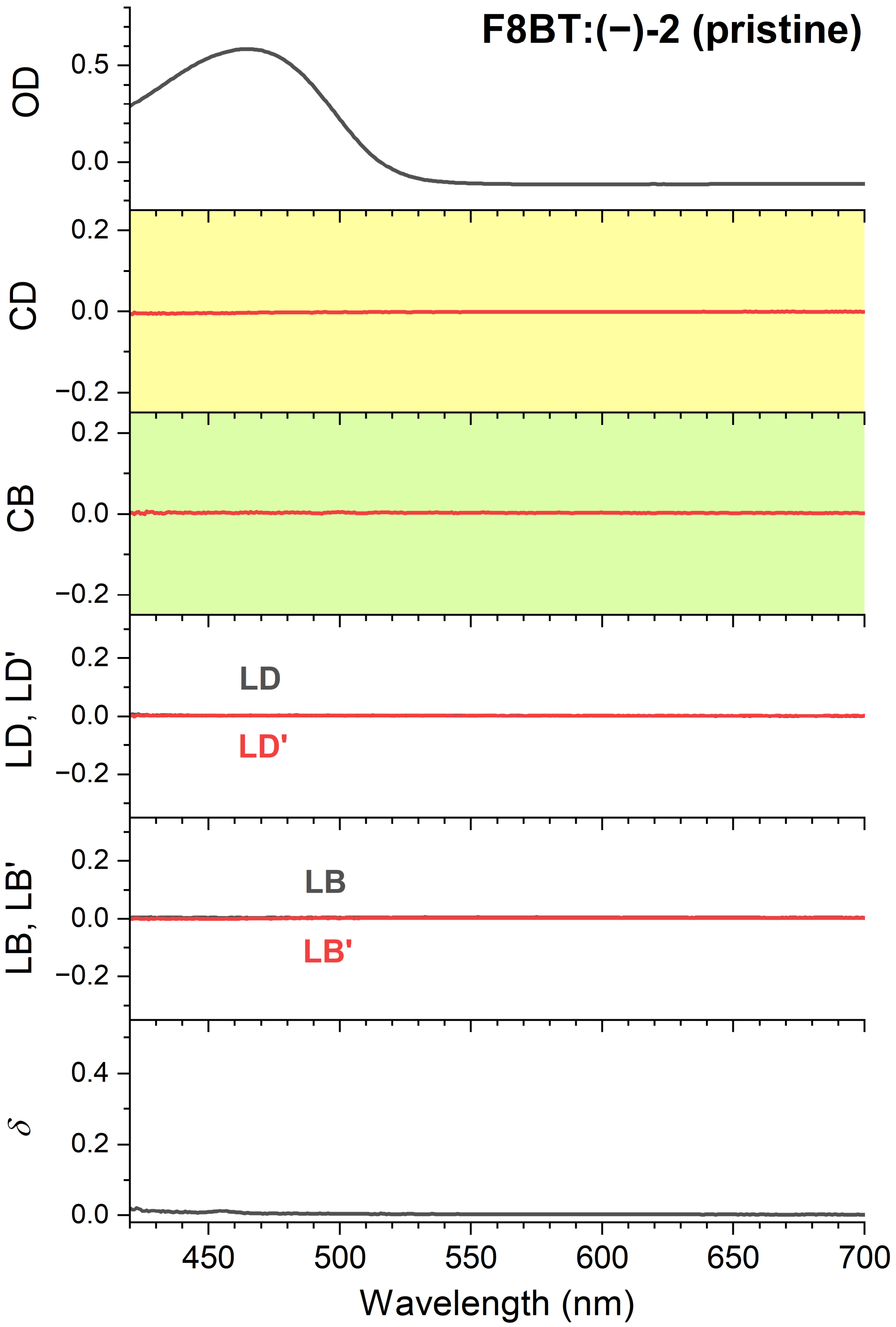

Supplementary Figure 6. Müller matrix spectra of a pristine F8BT:(−)-2 thin film. OD, CD (yellow background), CB (green background), LD/LD’ and LB/LB’ response as well as the depolarisation parameter δ (from top to bottom).

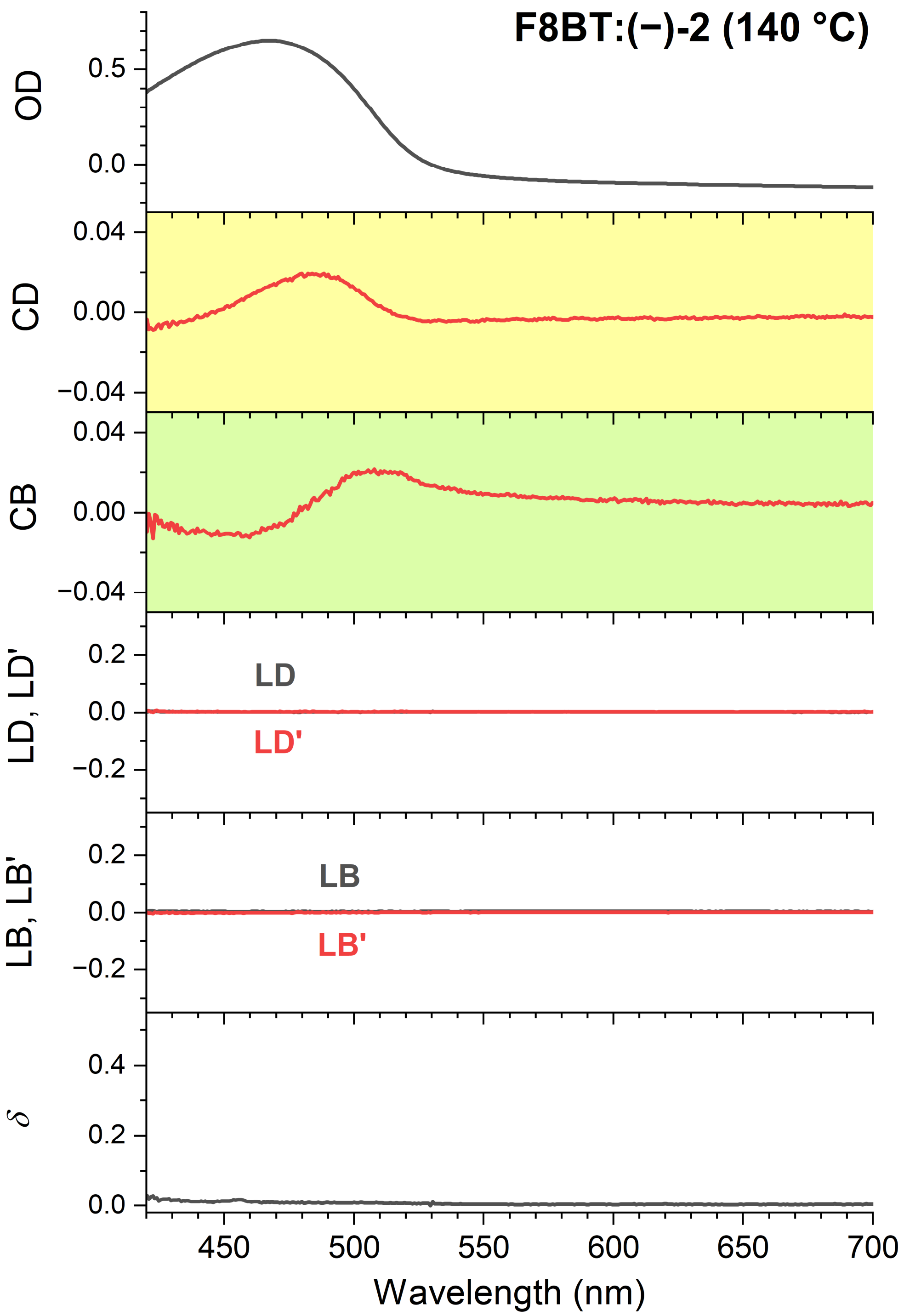

Supplementary Figure 7. Müller matrix spectra of an F8BT:(−)-2 thin film annealed at 140 °C. OD, CD (yellow background), CB (green background), LD/LD' and LB/LB' response as well as the depolarisation parameter δ (from top to bottom).

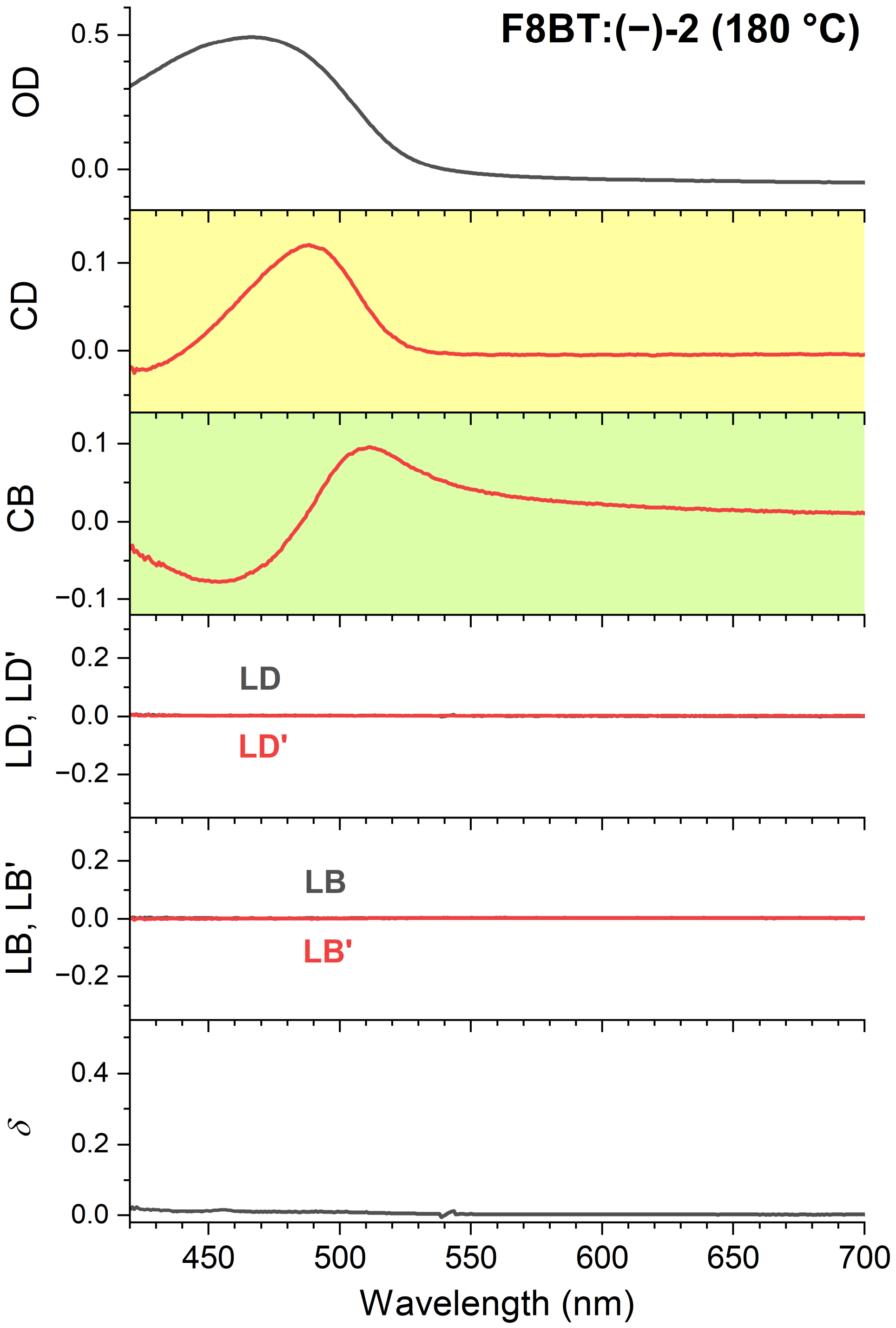

Supplementary Figure 8. Müller matrix spectra of an F8BT:(−)-2 thin film annealed at 180 °C. OD, CD (yellow background), CB (green background), LD/LD’ and LB/LB’ response as well as the depolarisation parameter δ (from top to bottom).

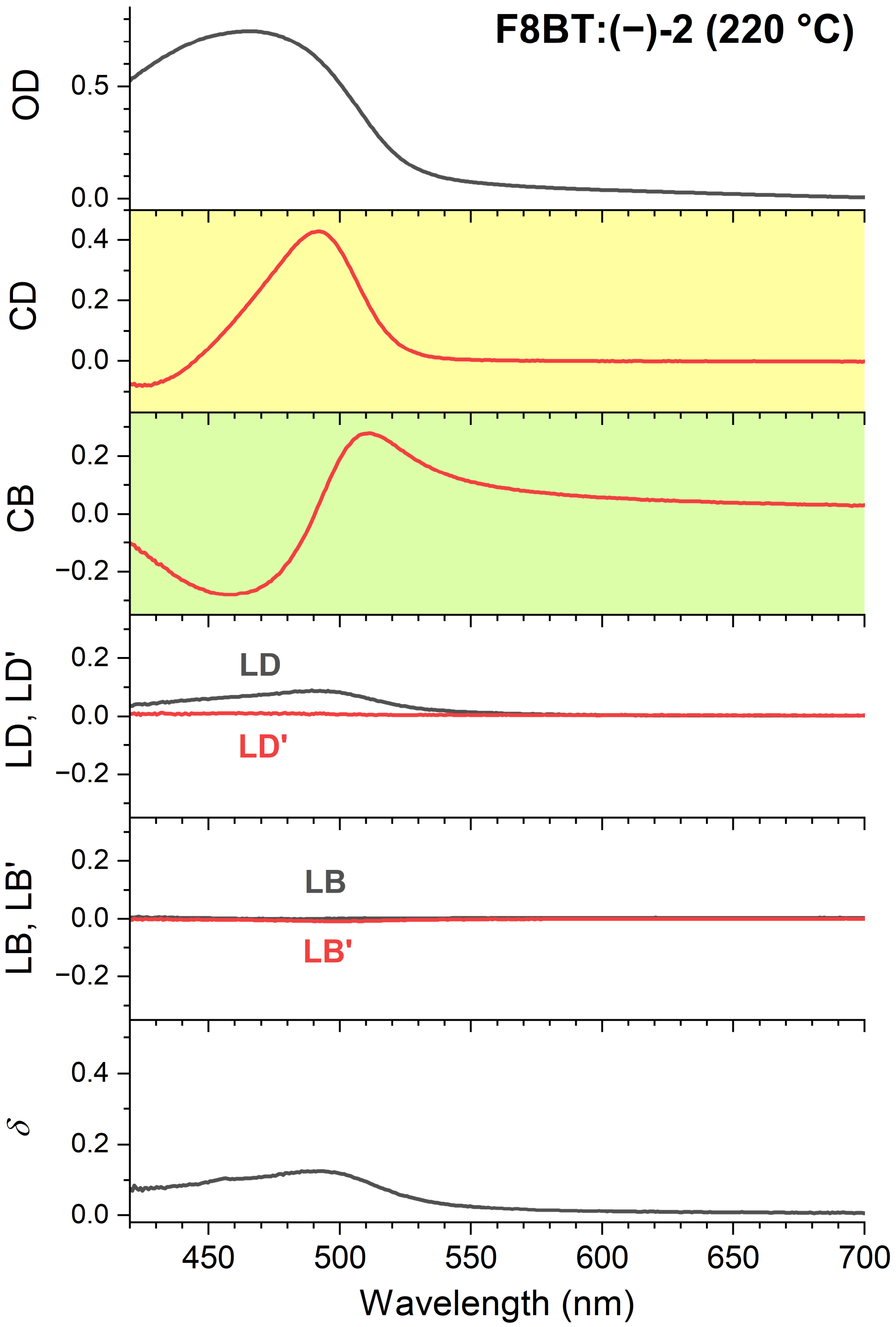

Supplementary Figure 9. Müller matrix spectra of an F8BT:(−)-2 thin film annealed at 220 °C. OD, CD (yellow background), CB (green background), LD/LD' and LB/LB' response as well as the depolarisation parameter δ (from top to bottom).

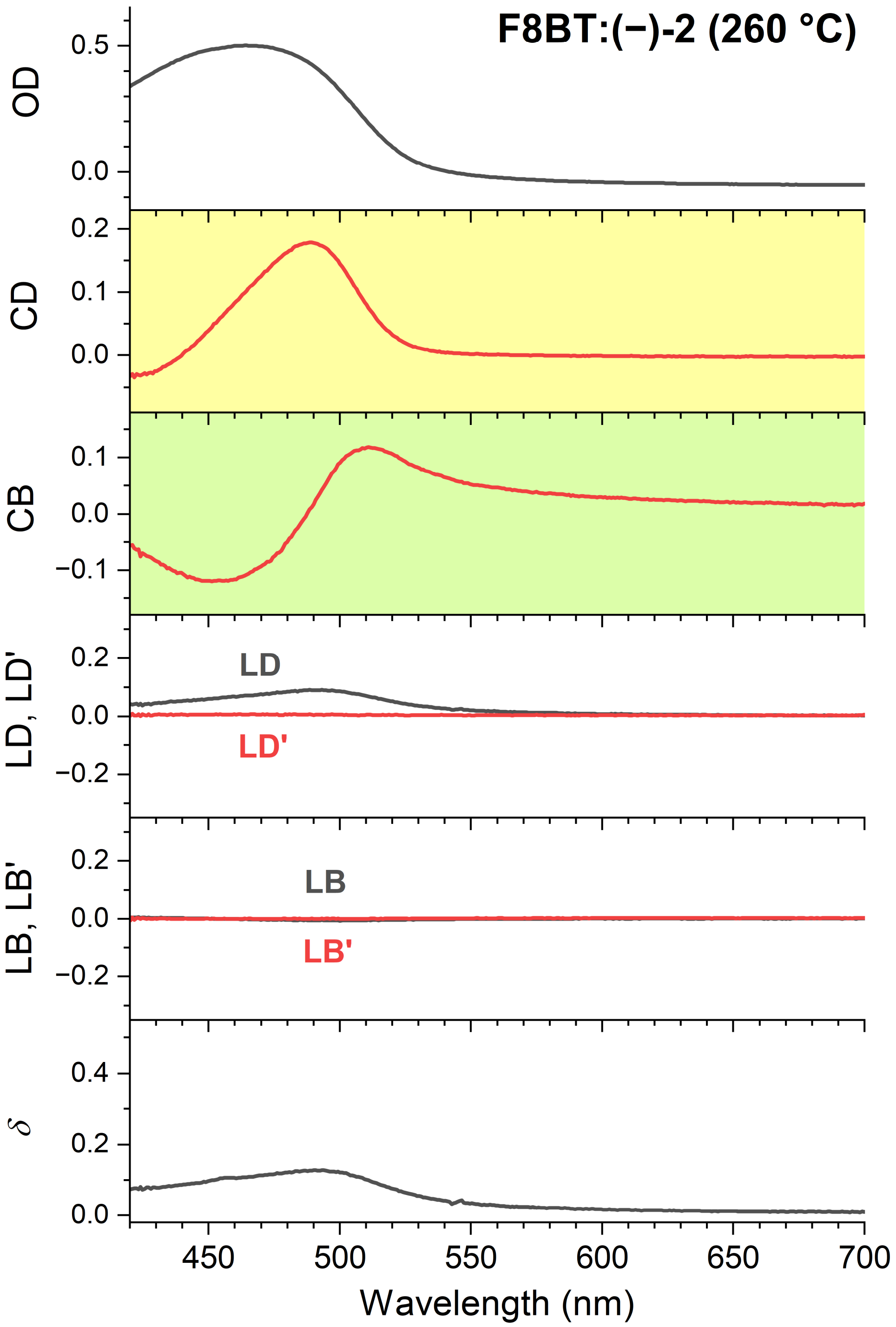

Supplementary Figure 10. Müller matrix spectra of an F8BT:(−)-2 thin film annealed at 260 °C. OD, CD (yellow background), CB (green background), LD/LD′ and LB/LB′ response as well as the depolarisation parameter δ (from top to bottom).

Supplementary Note 2.
Invariance of CD images under sample rotation and flipping

Supplementary Fig. 11 shows that upon turning an F8BT:(+)-2 thin film sample, there are virtually no changes in the CD response and the dissymmetry factor g_{abs} recorded in the CD images. The stepwise 90° rotation can be nicely recognised by following the rotation of the red filamentary structures of different shape. The g_{abs} distributions, which can be fitted by a sum of two Gaussian functions, are identical within error limits. In addition, the area-integrated spectra for OD_L, OD_R, OD_L-OD_R and g_{abs} are virtually identical. Therefore, one can safely conclude that for these chiral thin films, CD and g_{abs} are invariant under rotation down to the micrometre length scale.

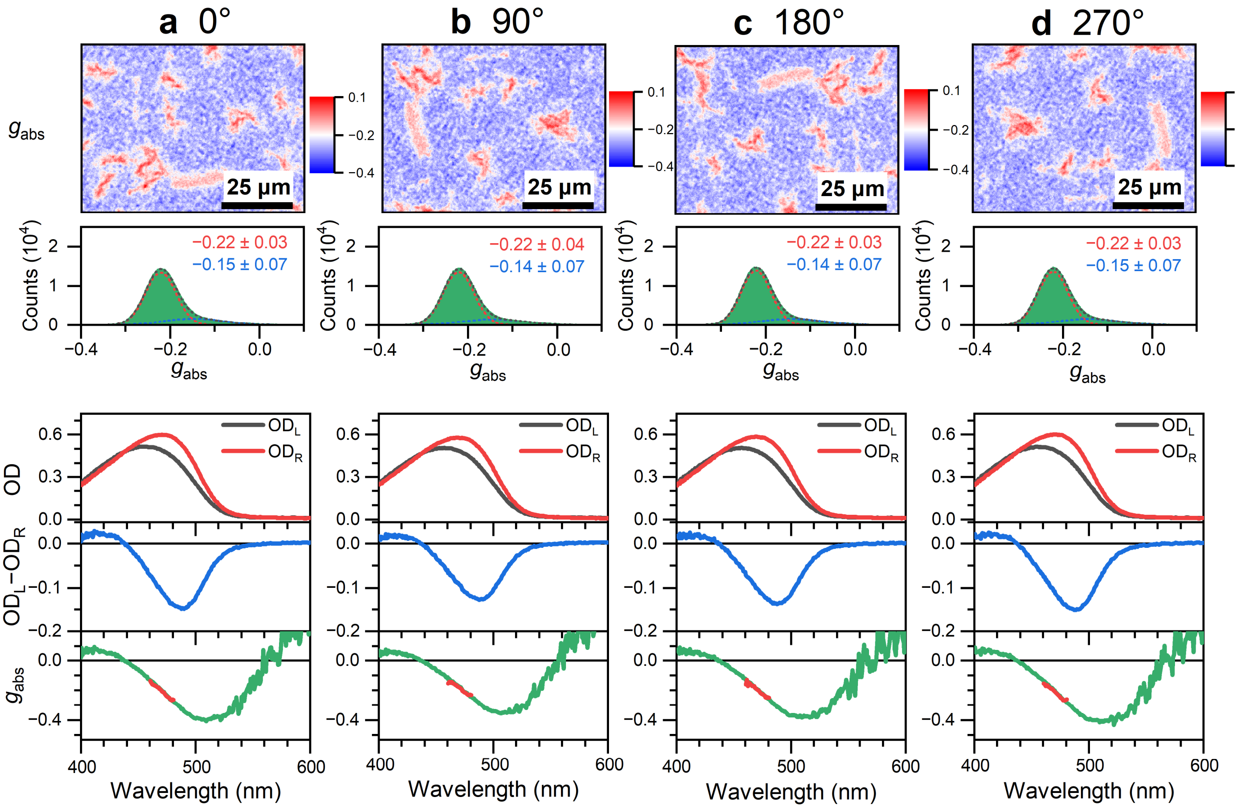

Supplementary Figure 11. Invariance of a CD image under clockwise rotation for an F8BT:(+)-2 thin film sample annealed at 180 °C for 15 min. a Microscope image (80 × 60 μm^2, length of scale bar: 25 μm) showing the dissymmetry parameter g_{abs} of a F8BT:(+)-2 thin film (top) with the distribution of g_{abs} values (middle) including a fit of two Gaussian functions (red and blue dashed lines, with the sum shown as a black dashed line), determined over the entire field of view, and corresponding spectra integrated over the entire field of view (210 × 160 μm^2, three panels at the bottom) displaying OD_L (black line) and OD_R (red line), the CD spectrum (OD_L-OD_R, blue line) and the g_{abs} spectrum (green line), with the red line indicating the spectral region selected by the bandpass filter (470 nm, FWHM 10 nm) used for CD imaging. **b–d** Same as in panel a, but sample manually rotated clockwise in 90° steps.

Supplementary Fig. 12 shows the behaviour upon flipping of the sample for another F8BT:(+)-2 thin film. The arrow-like scratch on the left side of the CD images serves as an orientation mark. We focus on the elongated extended blue region in the top right quadrant of panel a. After flipping, this blue

region now appears in the bottom right quadrant of the image. The g_{abs} distributions are identical within error limits, and the area-integrated spectra for OD_L, OD_R, OD_L−OD_R and g_{abs} are similar. One can therefore exclude contributions on the micrometre scale resulting from a combination of linear dichroism and linear birefringence ("LDLB effect") in combination with any possible anisotropies of the CD microscopy setup.

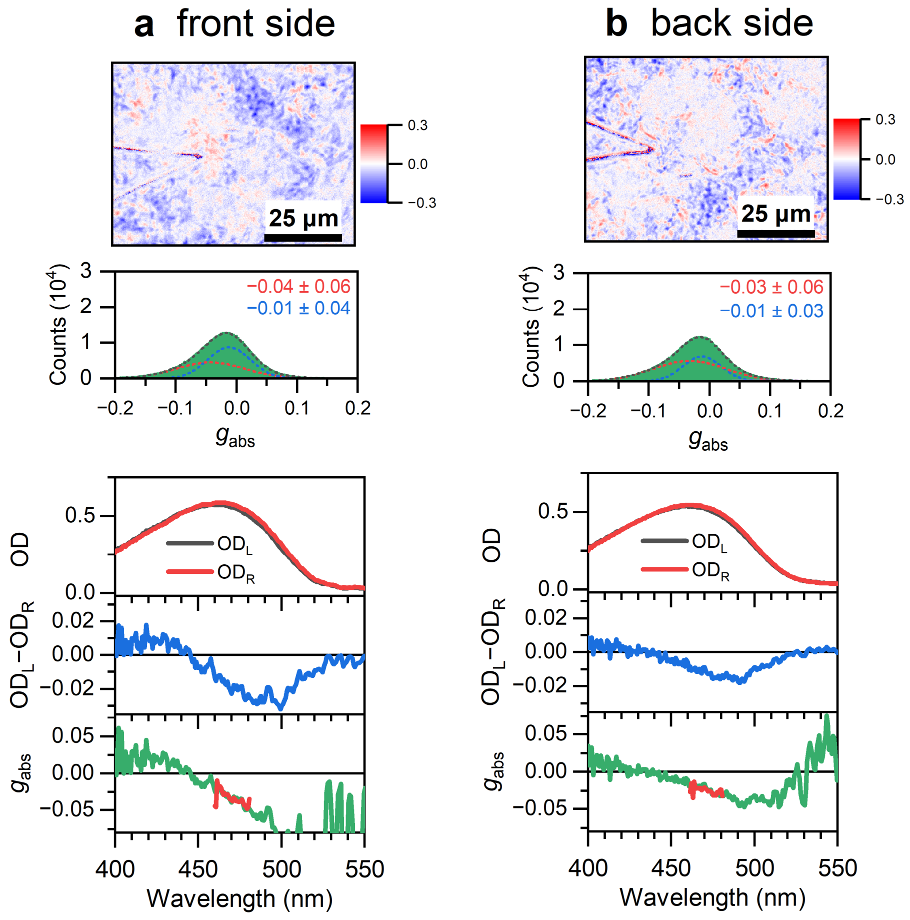

Supplementary Figure 12. Invariance of a CD image under flipping for an F8BT:(+)-2 thin film sample annealed at 180 °C for 60 min. a Microscope image (80 × 60 μm^2, length of scale bar: 25 μm) showing the dissymmetry parameter g_{abs} of a F8BT:(+)-2 thin film (top) with the distribution of g_{abs} values (middle) including a fit of two Gaussian functions (red and blue dashed lines, with the sum shown as a black dashed line), determined over the entire field of view, and corresponding spectra integrated over the entire field of view (210 × 160 μm^2, three panels at the bottom) displaying OD_L (black line) and OD_R (red line), the CD spectrum (OD_L−OD_R, blue line) and the g_{abs} spectrum (green line), with the red line indicating the spectral region selected by the bandpass filter (470 nm, FWHM 10 nm) used for CD imaging. **b** Same as in panel a, but for the flipped sample. The arrow-like scratch on the left side of the CD images serves as an orientation mark. Note that, because of the extended heat treatment of this thin film, its absolute CD response is considerably lower than for the thin film shown in Supplementary Fig. 11.

Supplementary Note 3.
Additional transient absorption and TrCD spectra of thin films

Supplementary Fig. 13 summarises transient absorption and TrCD spectra for an F8BT:(+)-2 thin film (panel a) and an F8BT:(−)-2 thin film (panel b), which were selected from the contour plot data of Fig. 6 (main manuscript).

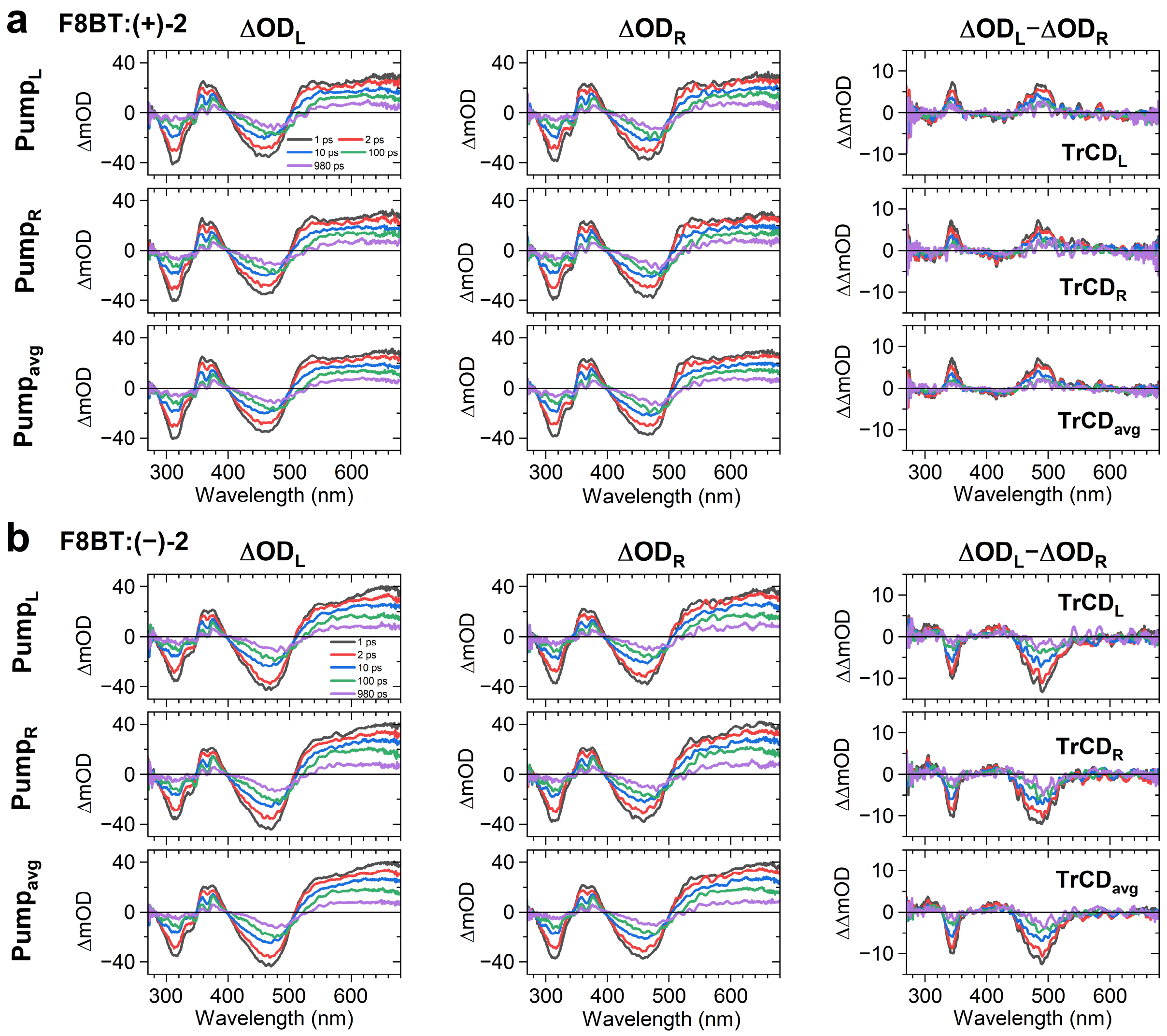

Supplementary Figure 13. Transient spectra at selected pump–probe time delays between 1 ps and 980 ps for chiral F8BT:(+)-2 and F8BT:(−)-2 thin film blends annealed at 220 °C. a Transient spectra of the F8BT:(+)-2 blend for excitation by an LCP pump pulse (first row) or an RCP pump pulse (second row), as well as for the average of LCP and RCP excitation representing random polarisation (third row). The first two columns show the transient absorption spectra probed by an LCP supercontinuum (ΔOD$_L$) and an RCP supercontinuum (ΔOD$_R$), respectively, and the third column displays the resulting TrCD spectra, which correspond to the difference ΔOD$_L$−ΔOD$_R$. **b** Same as in panel a, but for the F8BT:(−)-2 blend. Pump wavelength: 400 nm.

Supplementary Note 4.
NMR and mass spectra

Supplementary Figs. 14–16 contain the ^{1}H and ^{13}C NMR spectra as well as the ESI mass spectrum of compound (+)-2.

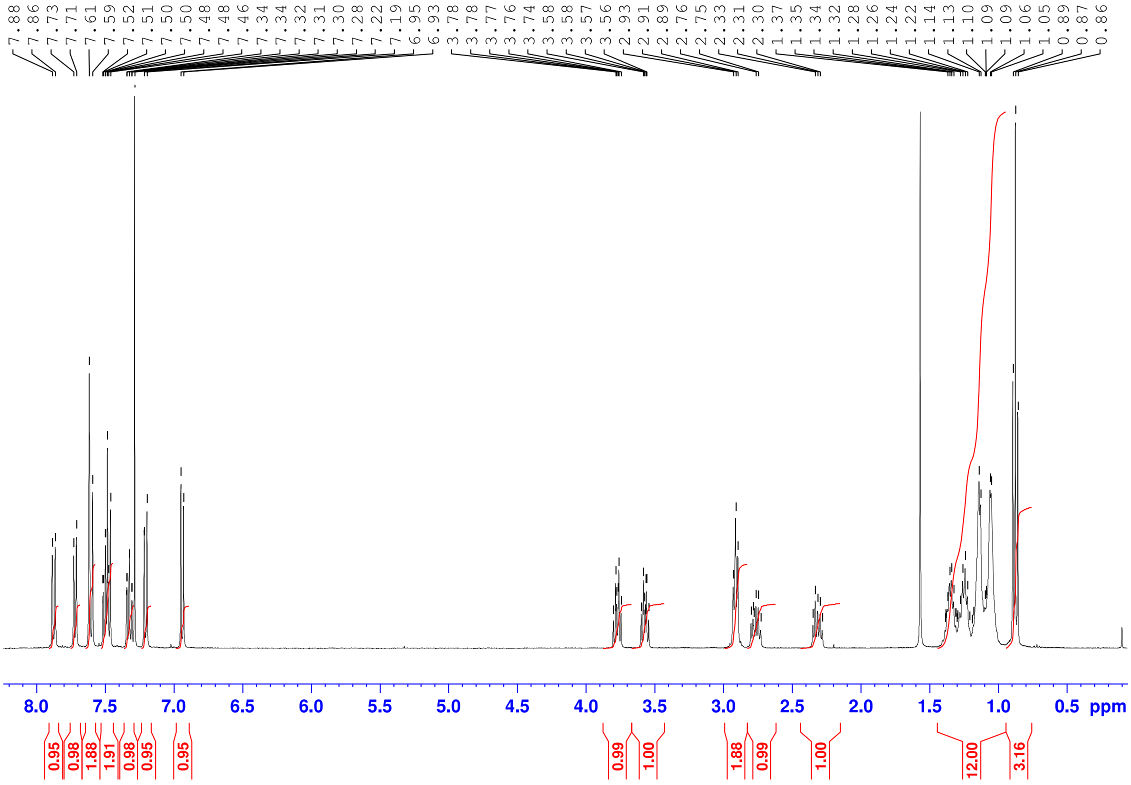

Supplementary Figure 14. ^{1}H NMR spectrum ($CDCl_3$, 400.14 MHz) of compound (+)-2.

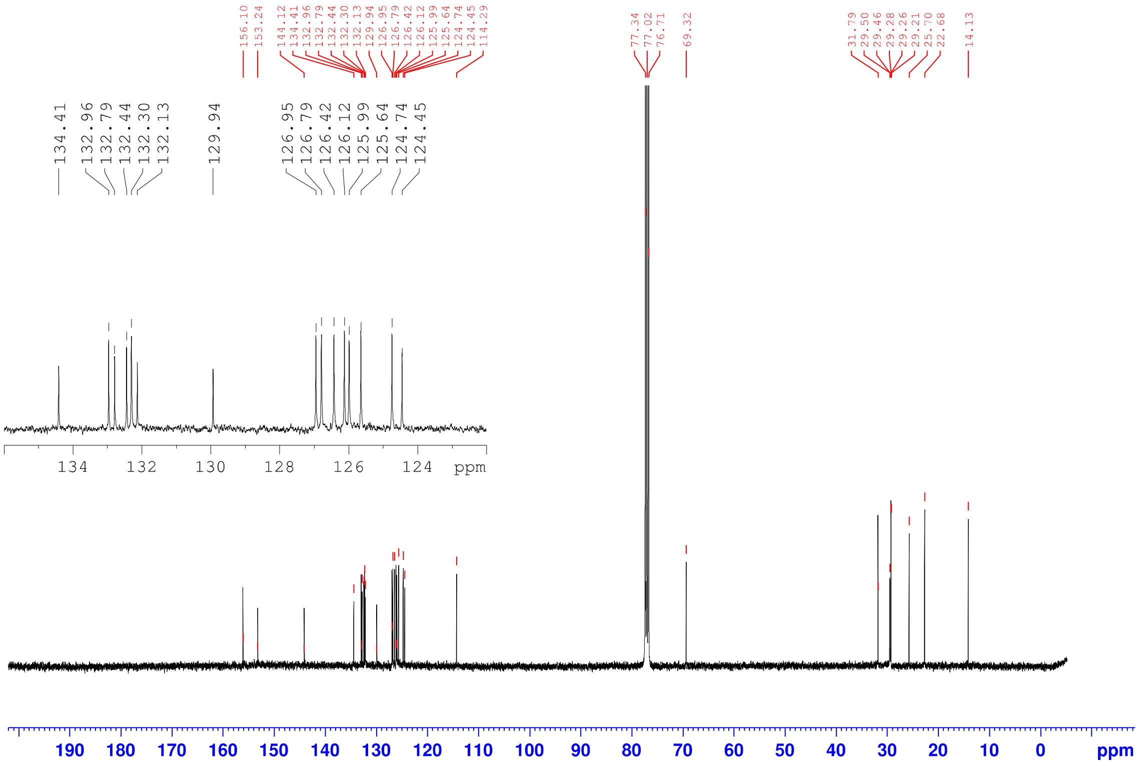

Supplementary Figure 15. ^{13}C **NMR spectrum ($CDCl_3$, 100.62 MHz) of compound (+)-2.**

CENTRE COMMUN DE SPECTROMETRIE DE MASSE

Analysis Info

Analysis Name	Impact2_240213_02_JCM2067.d		
Method	Tune_pos_Standard.m	Acquisition Date	2/13/2024 10:03:02 AM
Comment		Instrument / Ser#	impact II 1825265.10081

Acquisition Parameter

Source Type	ESI	Ion Polarity	Positive	Set Nebulizer	0.3 Bar
Focus	Active	Set Capillary	4500 V	Set Dry Heater	200 °C
Scan Begin	50 m/z	Set End Plate Offset	-500 V	Set Dry Gas	4.0 l/min
Scan End	2000 m/z	Set Collision Cell RF	1500.0 Vpp	Set Divert Valve	Source

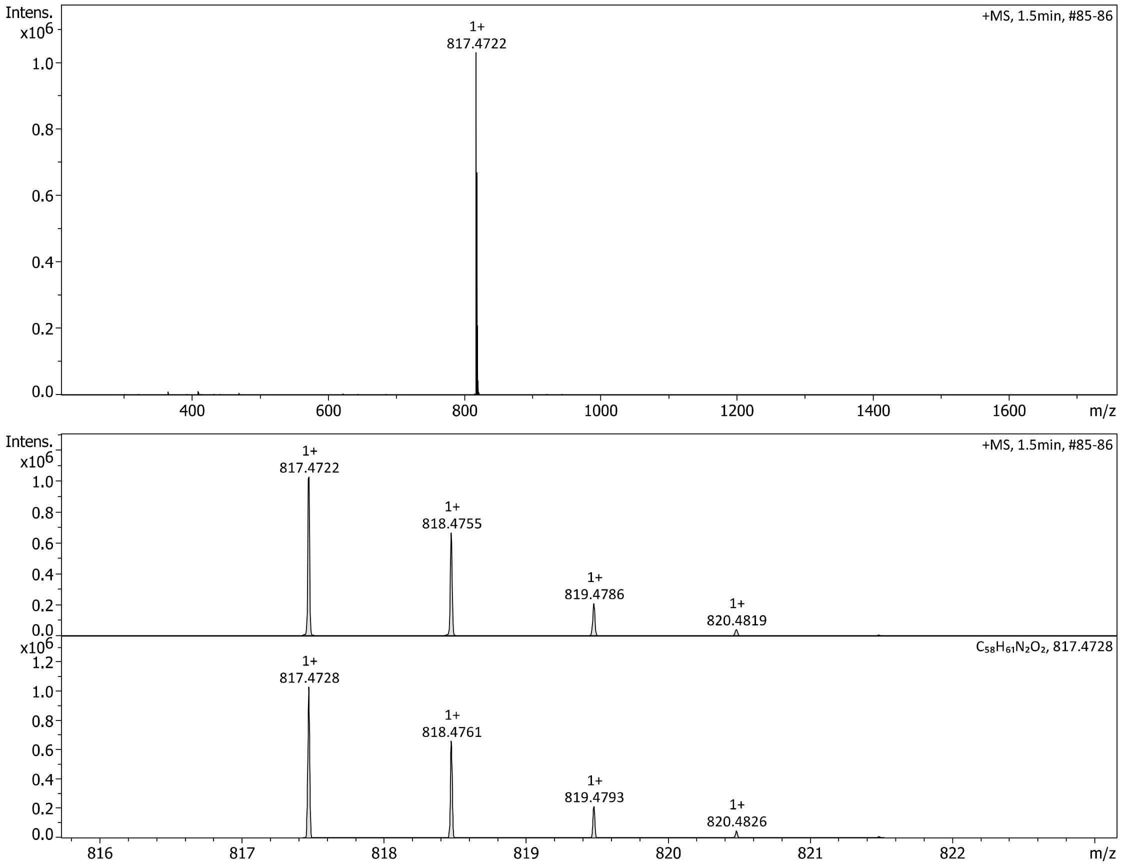

Meas. m/z	Ion Formula	m/z	Sum Formula	err [ppm]	mSigma	Adduct	z
817.4722	C58H61N2O2	817.4728	C58H60N2O2	0.7	4.4	M+H	1+

Bruker Compass DataAnalysis 6.1 Page 1 of 1

Supplementary Figure 16. ESI mass spectrum of compound (+)-2.

Supplementary Note 5.
Coherent acoustic phonon dynamics of thin films

Supplementary Fig. 17 shows representative transient absorption kinetics averaged over the wavelength range 490–520 nm for a pristine F8BT film and three different F8BT:(+)-2 and F8BT:(−)-2 blends. These kinetic traces were employed to determine the film thickness using picosecond ultrasonics. From each fit, the coherent acoustic phonon oscillation period τ_a was extracted. The thickness d was determined as $d = 0.25 \cdot \tau_a \cdot c_L$, where c_L is the known longitudinal sound velocity of benzo[2,1,3]thiadiazol-based copolymers of 2490 m s^{-1}. The thickness of all films is between 175 nm and 287 nm, and thus in a range, where the dissymmetry parameter g_{abs} for the chiral thin films reaches a saturation value.

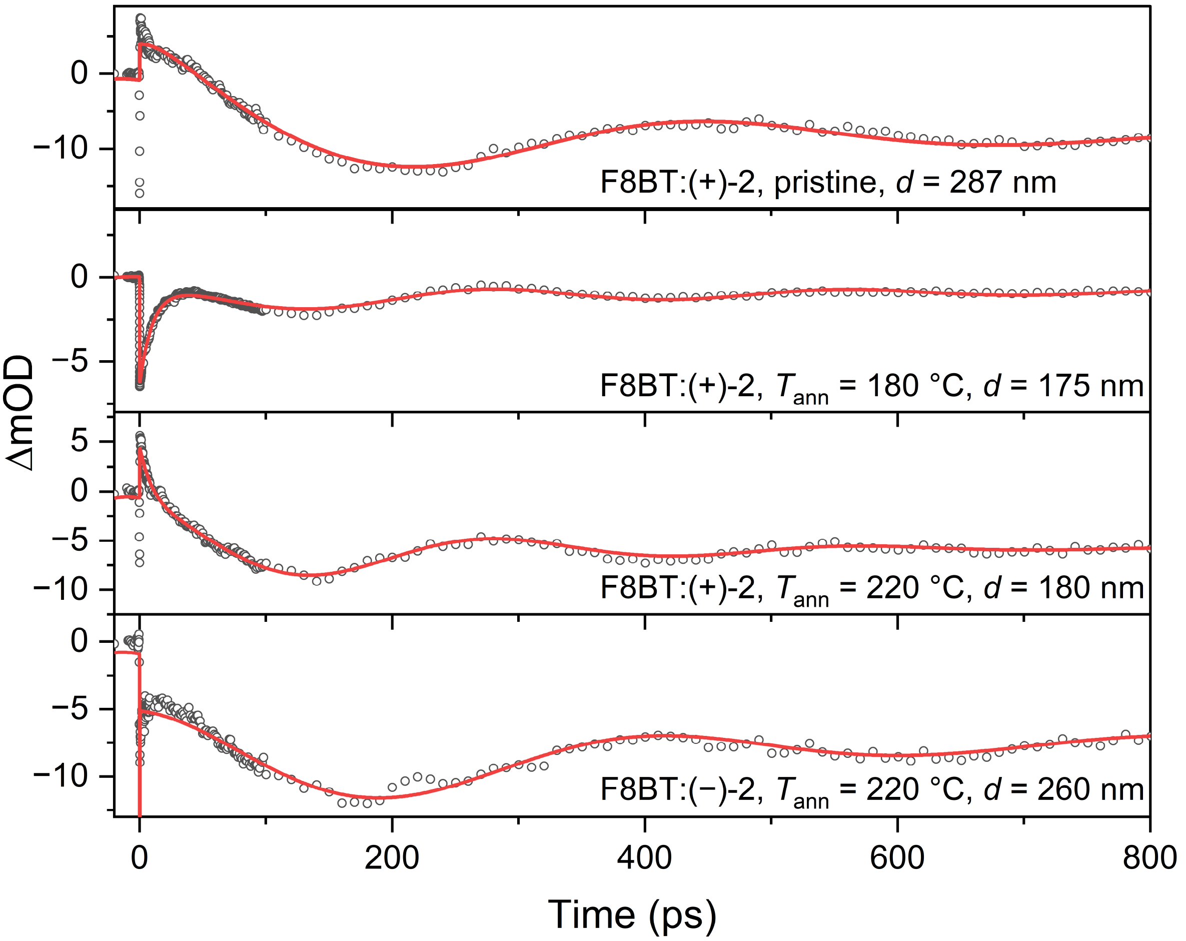

Supplementary Figure 17. Picosecond ultrasonics of F8BT, F8BT:(+)-2 and F8BT:(−)-2 thin films. The experimental data (open circles) are averages of 35 kinetic traces over the wavelength range 490–520 nm. The best fits (red lines) consist of two exponential decays, a step-function and a damped cosine function, all convolved with a Gaussian function. λ_{Pump} = 400 nm.

Supplementary Note 6.
Thickness dependence of g_{abs} for F8BT:(−)-2 thin films

Panel a of Supplementary Fig. 18 shows representative transient absorption kinetics from picosecond ultrasonics averaged over the wavelength range 490–520 nm for F8BT:(−)-2 blends. An analysis of the oscillations provided thickness values between 47 nm and 408 nm. Panels b and c show the thickness-dependent g_{abs} values of these films in an absolute and normalised representation, respectively, for the wavelengths 500 nm (black squares) and 350 nm (red circles). The dissymmetry parameter is close to zero below 100 nm thickness and rises sharply above until it levels off around 175 nm.

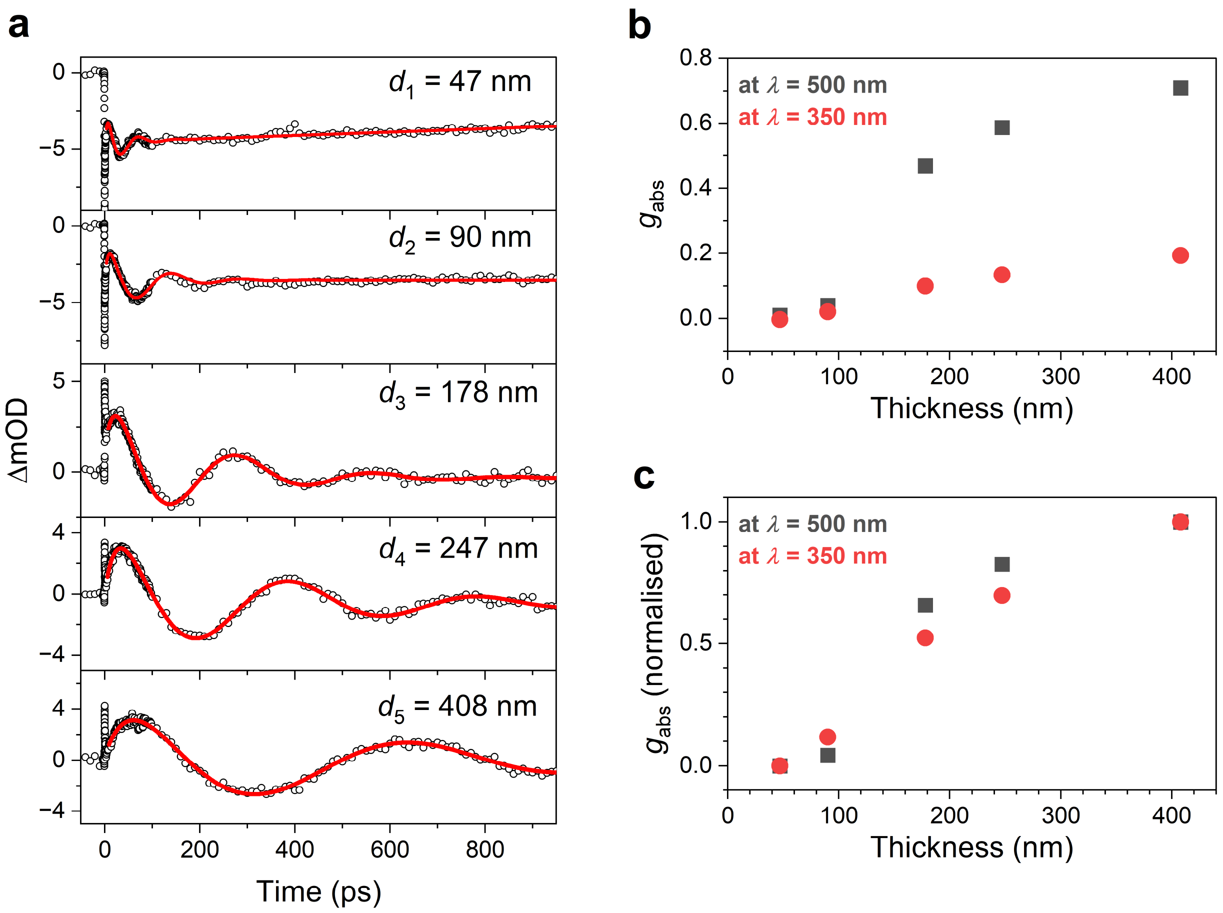

Supplementary Figure 18. Thickness dependence of g_{abs} for F8BT:(−)-2 thin films. a Transient absorption kinetics from picosecond ultrasonics (open circles) averaged over the wavelength range 490–520 nm (35 kinetic traces) including best fits (red lines) consisting of two exponential decays, a step-function and a damped cosine function, all convolved with a Gaussian function. λ_{Pump} = 400 nm. **b** Dissymmetry parameter g_{abs} of the thin films as a function of thickness, determined at the wavelengths 500 nm (black squares) and 350 nm (red circles). **c** Same as in panel b, but g_{abs} values at the lowest and highest film thickness normalised to 0 and 1, respectively, in both cases.
